# Supplementary material for: Non-C2-Symmetric Bis-Benzimidazolium Salt Applied in the Synthesis of Sterically Hindered Biaryls
Source: Molecules. 2021 Nov 5;26(21):6703. doi: 10.3390/molecules26216703 (PMC8588361; doi:10.3390/molecules26216703)

Supporting Information

Novel Amino Alcohol-Derived Bis-benzimidazolium Salt Catalyzed the Synthesis of Sterically Hindered Biaryls

Yen-Hsin Chen,[a] Shu-Jyun Huang,[a] Tung-Yu Hsu,[a] Pei-Yu Hung,[a] Dong-Sheng Lee,[a] and Ta-Jung Lu[a]*

[a] Department of Chemistry, National Chung Hsing University, Taichung, 402, Taiwan

Fax: 886-4-22862547; E-Mail: dslee@mail.nchu.edu.tw

Table of Contents

1. ^{1}H and ^{13}C-NMR Spectra of bis-benzimidazolium bromide **1**
   1.1. ^{1}H and ^{13}C-NMR Spectra of 3-methyl-2-(2-nitrophenylamino)-butan-1-ol (**S1**) S2
   1.2. ^{1}H and ^{13}C-NMR Spectra of 2-[(2-aminophenyl)amino]-3-methylbutan-1-ol (**S2**) S3
   1.3. ^{1}H and ^{13}C-NMR Spectra of 2-(1*H*-benzo[d]imidazol-1-yl-3-methylbutan-1-ol (**S3**) . S4
   1.4. ^{1}H and ^{13}C-NMR Spectra of 2-(1*H*-benzo[d]imidazol-1-yl)-3-methylbutyl 4-methyl benzenesulfonate (**S4**) S4
   1.5. ^{1}H and ^{13}C-NMR Spectra of 1,1'-(3-methylbutane-1,2-diyl)bis(1*H*-benzo[d]imidazole) (**2**) S4
   1.6. ^{1}H and ^{13}C-NMR Spectra of 1,1'-(3-methylbutane-1,2-diyl)bis(3-benzyl-1H-benzo[d]imidazol-3-ium) bromide (**1**) S4
2. ^{1}H and ^{13}C-NMR Spectra of Suzuki–Miyaura Cross-Coupling Products **5** S11

^{1}H NMR (CDCl$_3$, 400 MHz) spectrum of compound **S1**

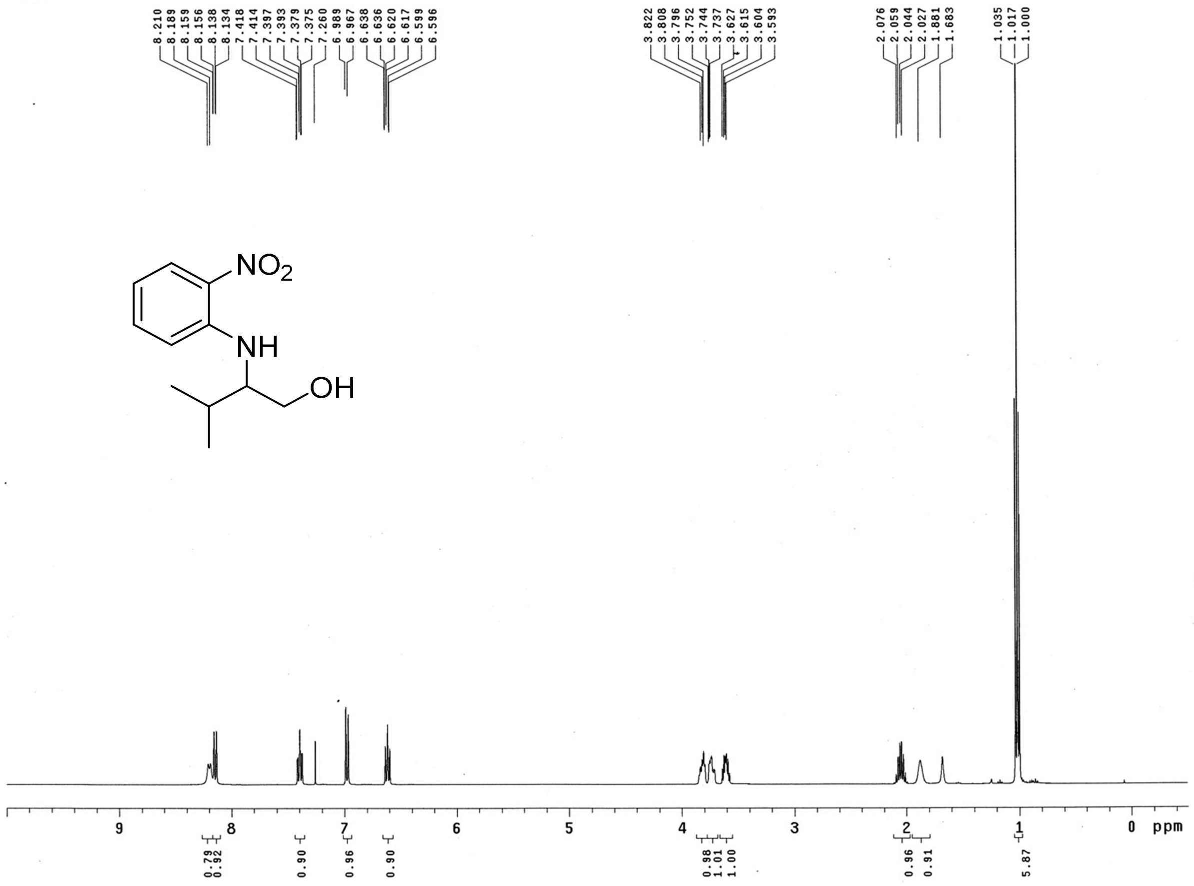

^{13}C NMR (CDCl$_3$, 100 MHz) spectrum of compound **S1**

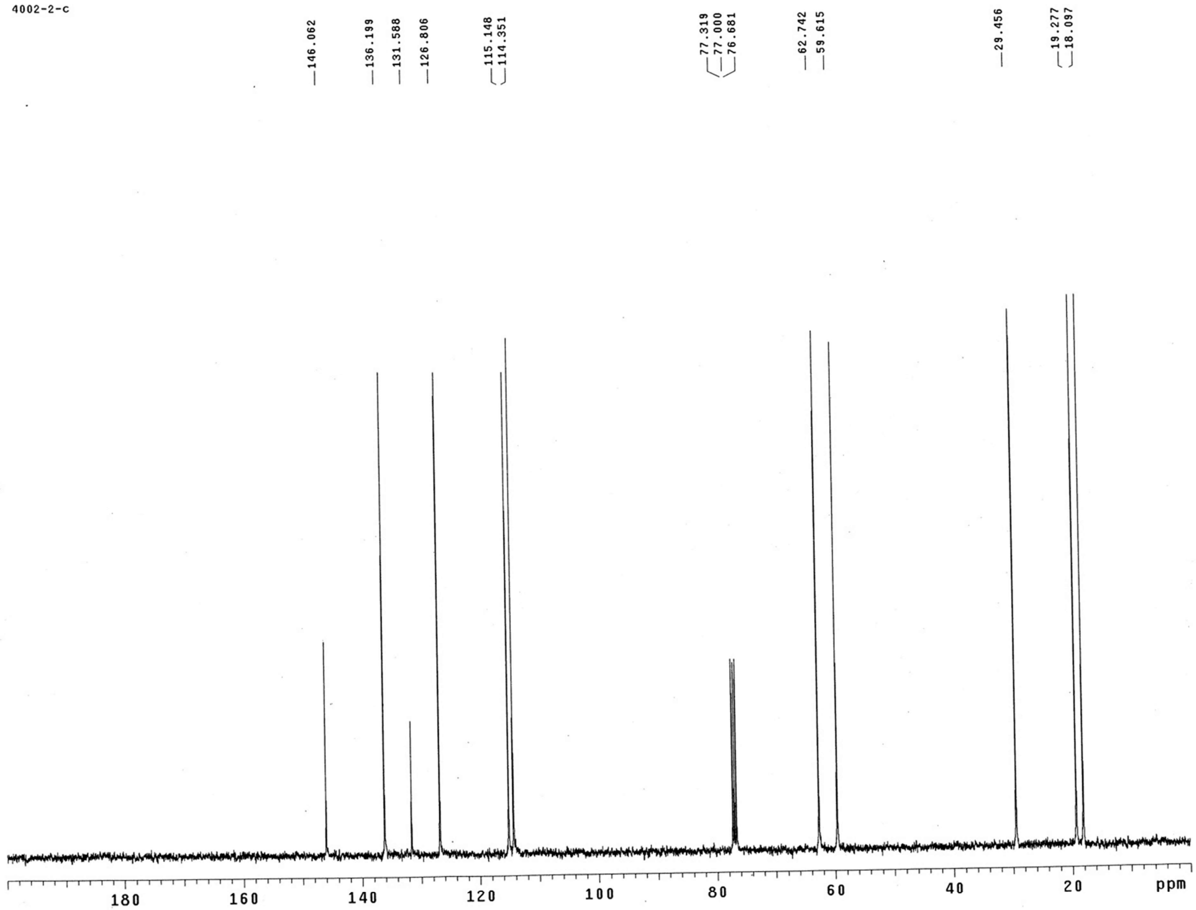

^{1}H NMR ($CDCl_3$, 400 MHz) spectrum of compound **S2**

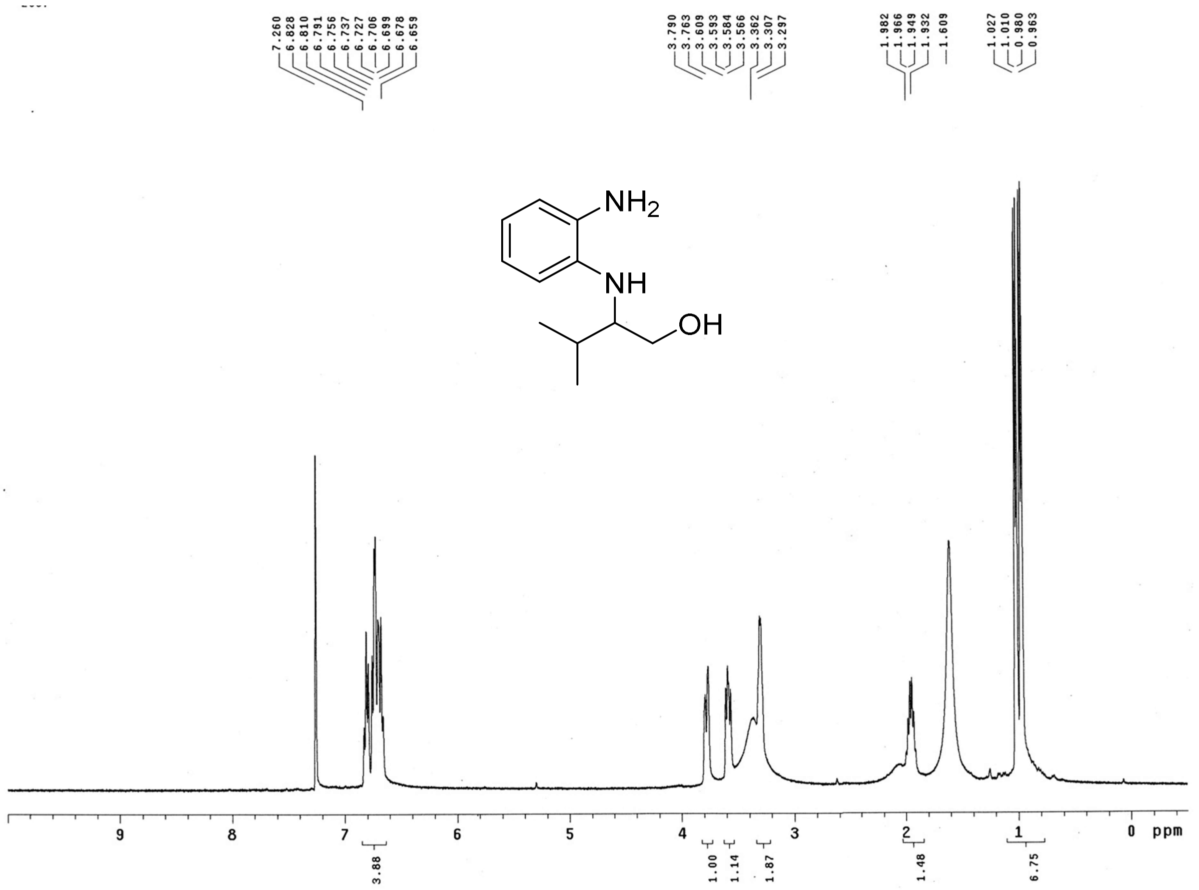

^{13}C NMR ($CDCl_3$, 100 MHz) spectrum of compound **S2**

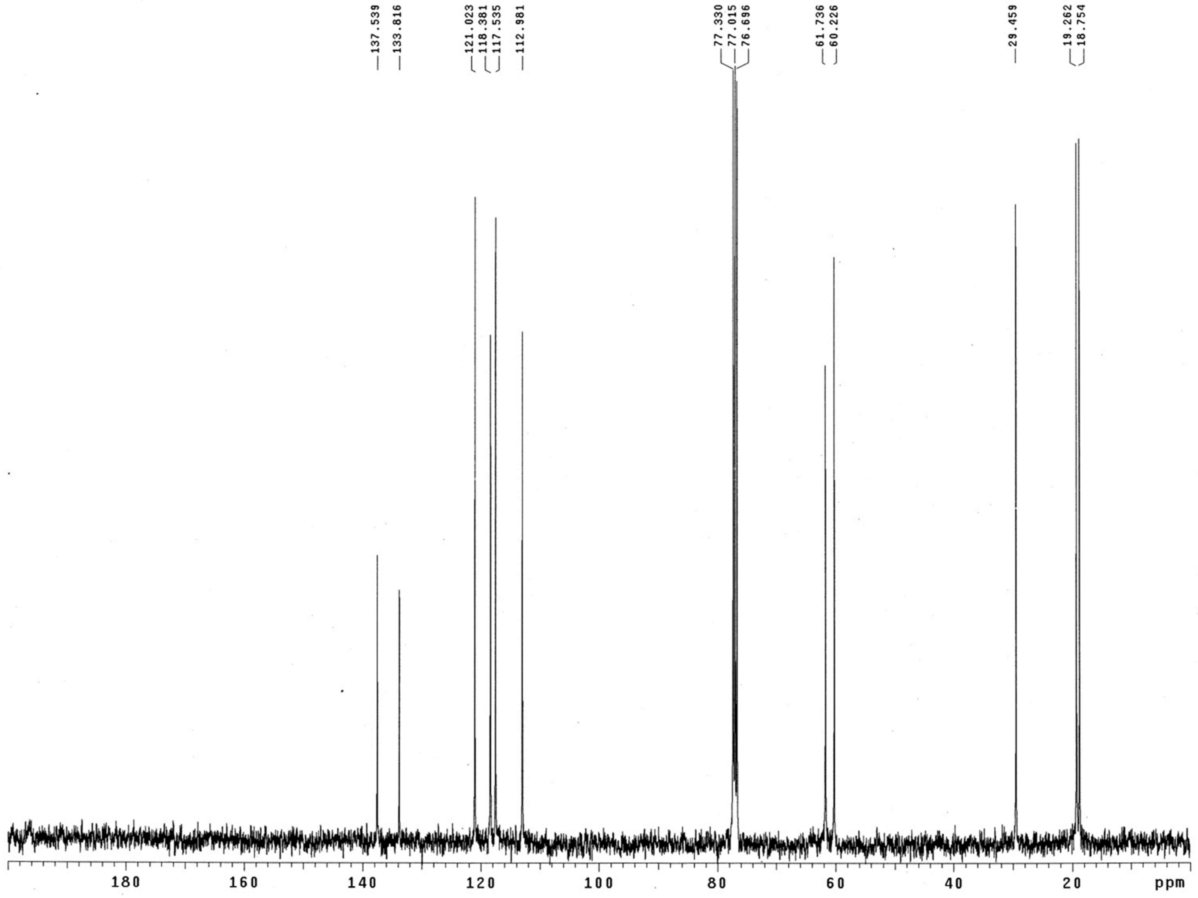

^{1}H NMR ($CDCl_3$, 400 MHz) spectrum of compound **S3**

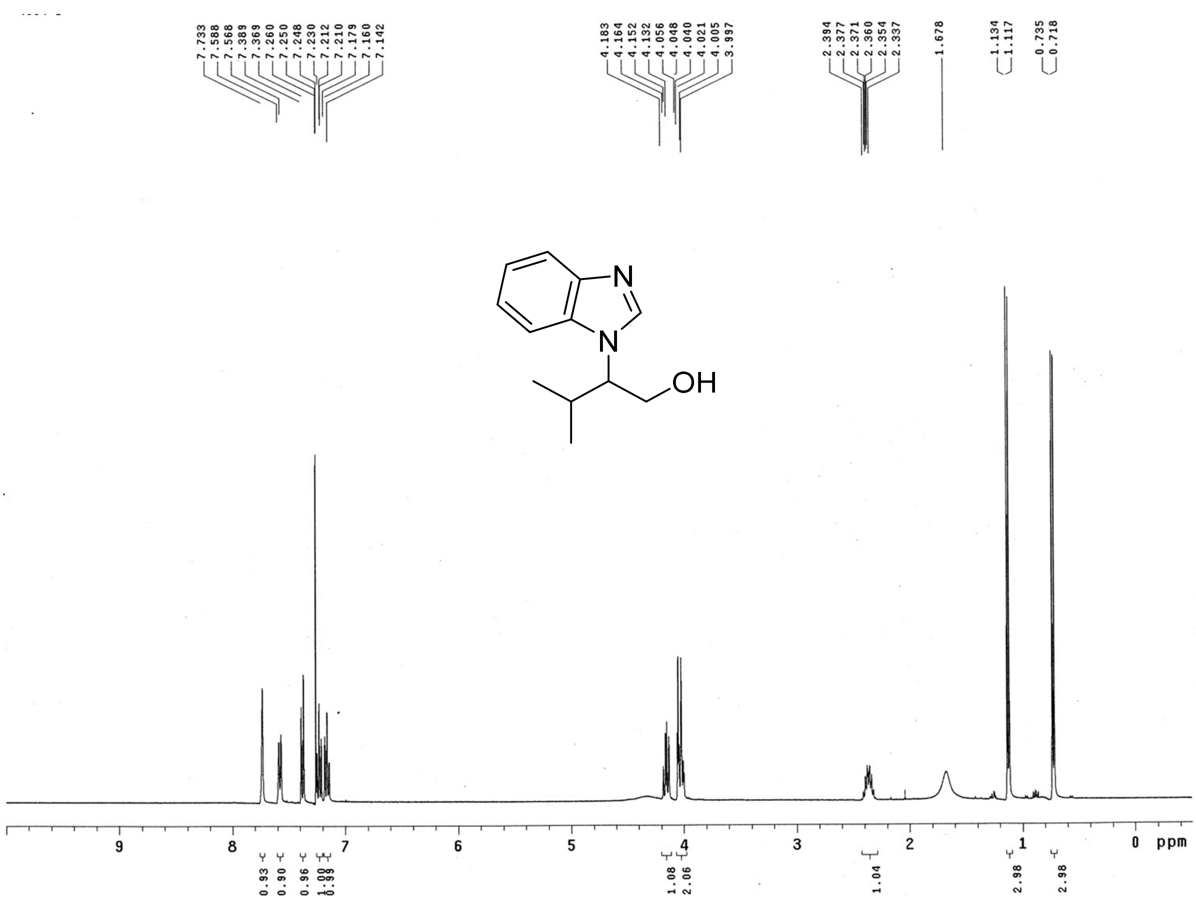

^{13}C NMR ($CDCl_3$, 100 MHz) spectrum of compound **S3**

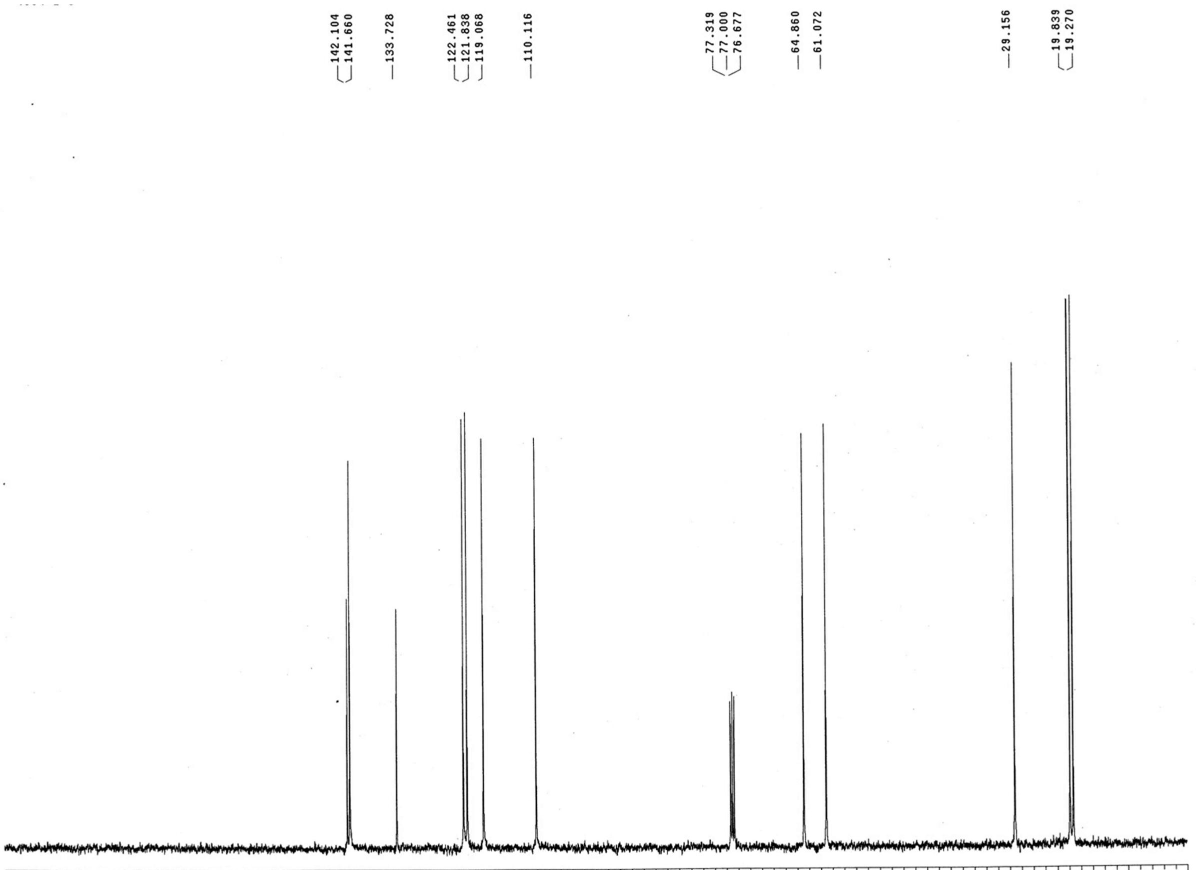

^{1}H NMR ($CDCl_3$, 400 MHz) spectrum of compound **S4**

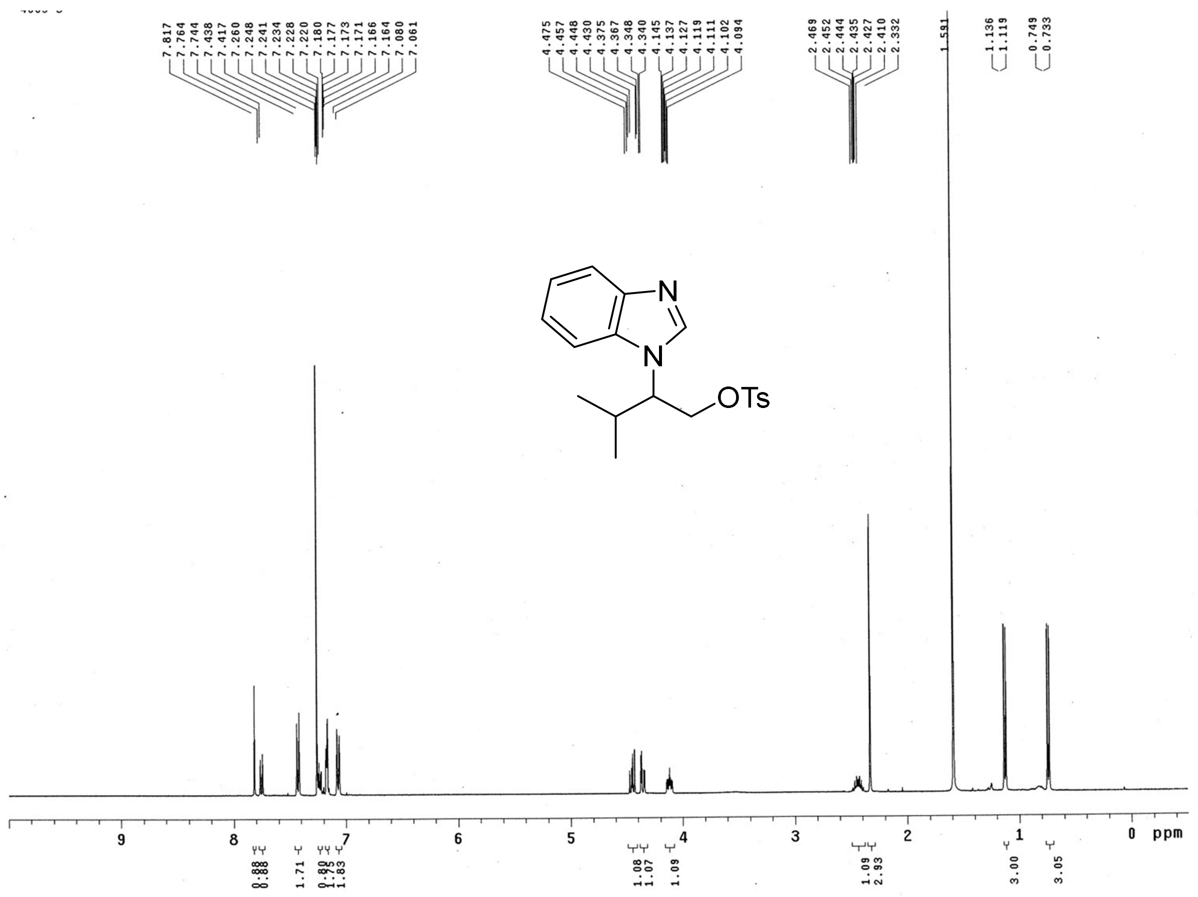

^{13}C NMR ($CDCl_3$, 100 MHz) spectrum of compound **S4**

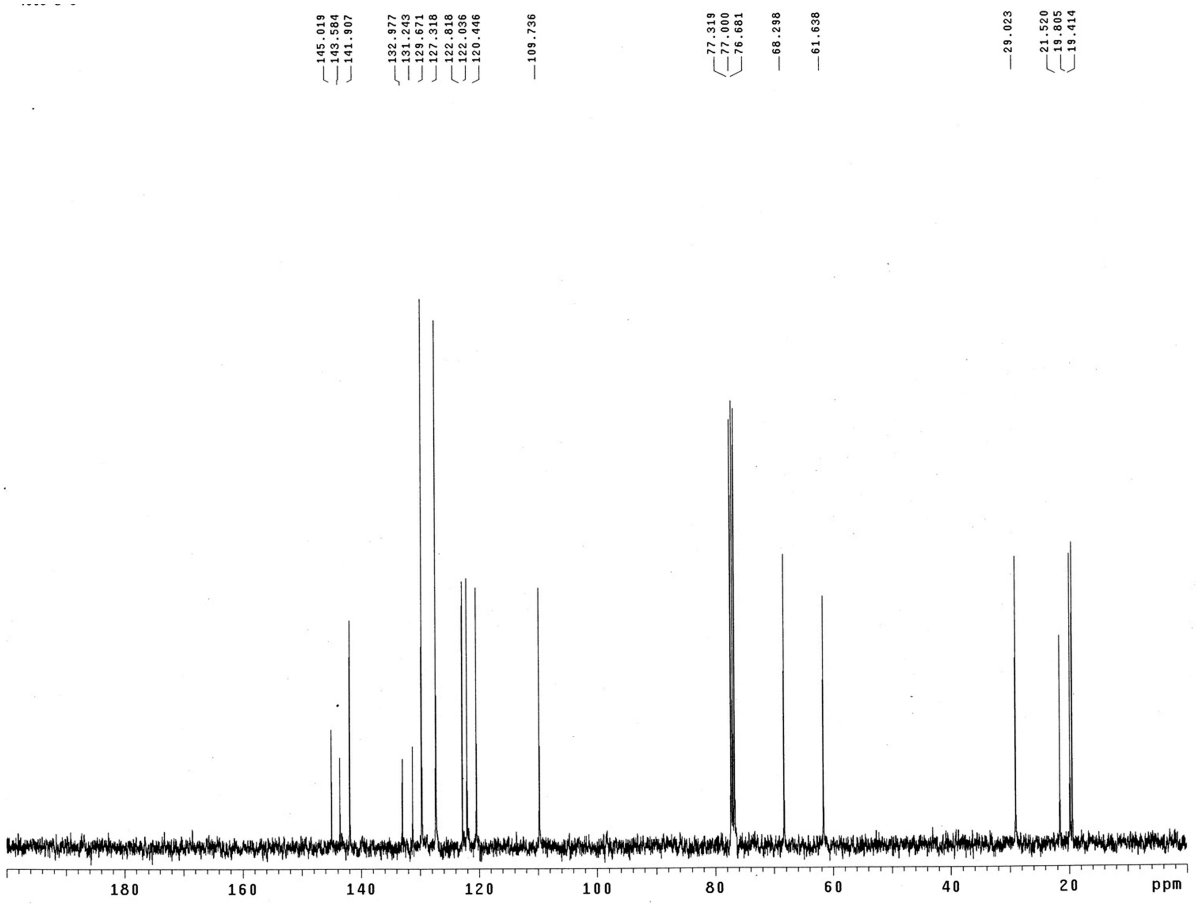

^{1}H NMR ($CDCl_3$, 400 MHz) spectrum of compound **2**

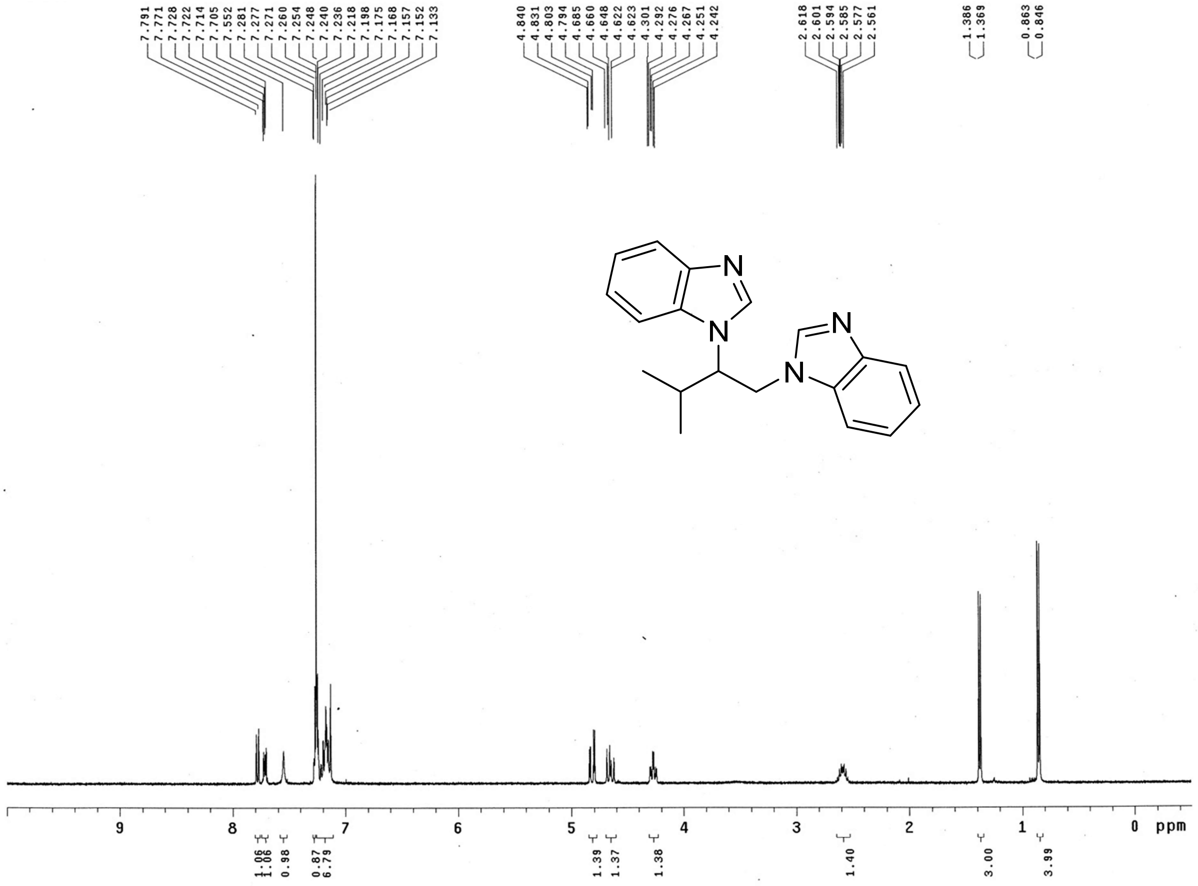

^{13}C NMR ($CDCl_3$, 100 MHz) spectrum of compound **2**

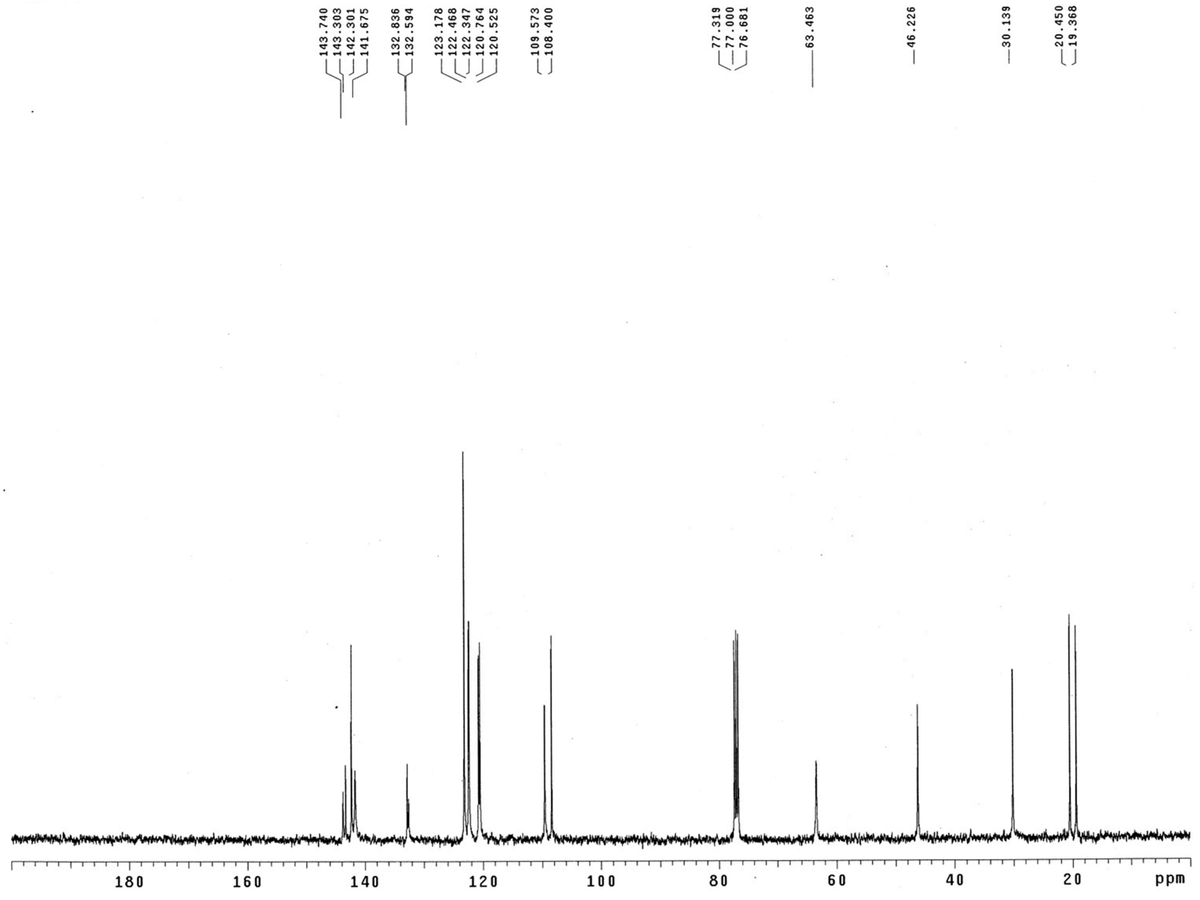

^{1}H NMR ($CDCl_3$, 400 MHz) spectrum of compound **1**

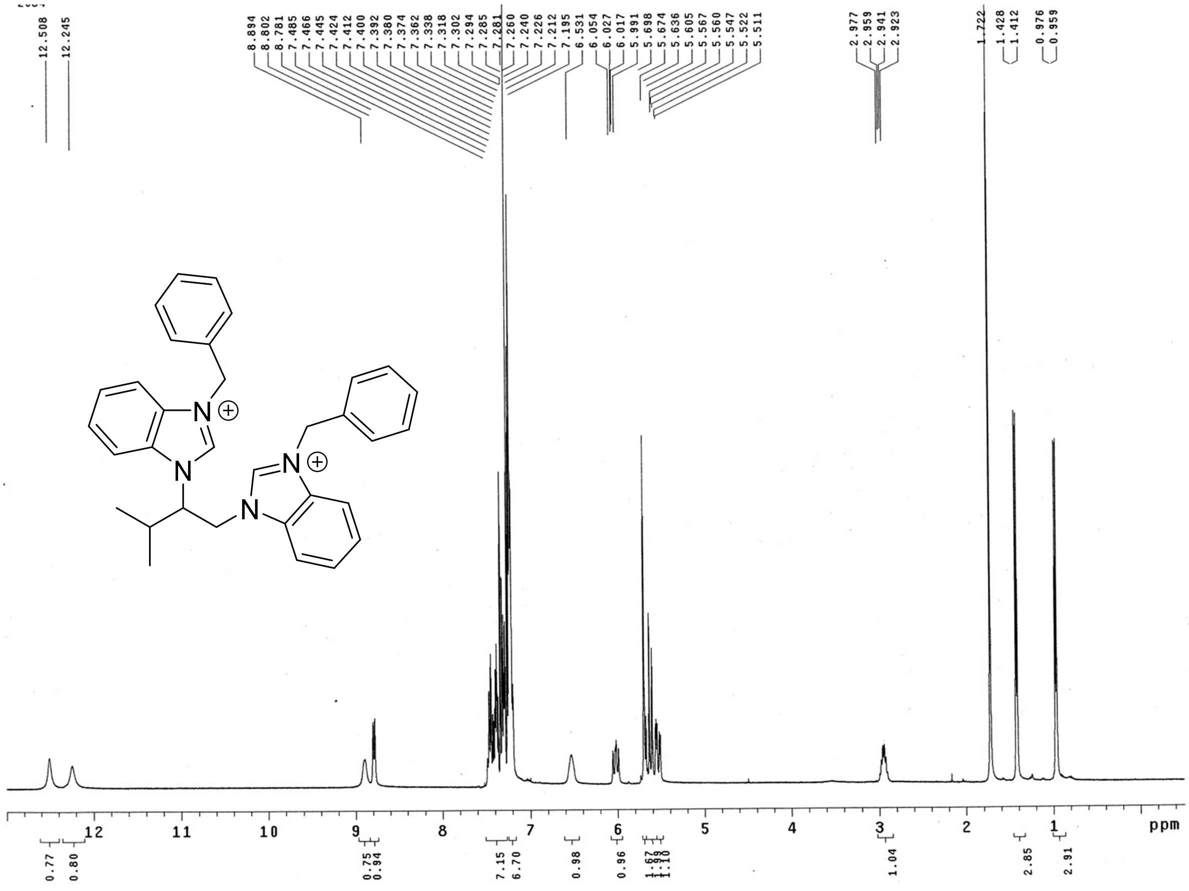

^{13}C NMR ($CDCl_3$, 100 MHz) spectrum of compound **1**

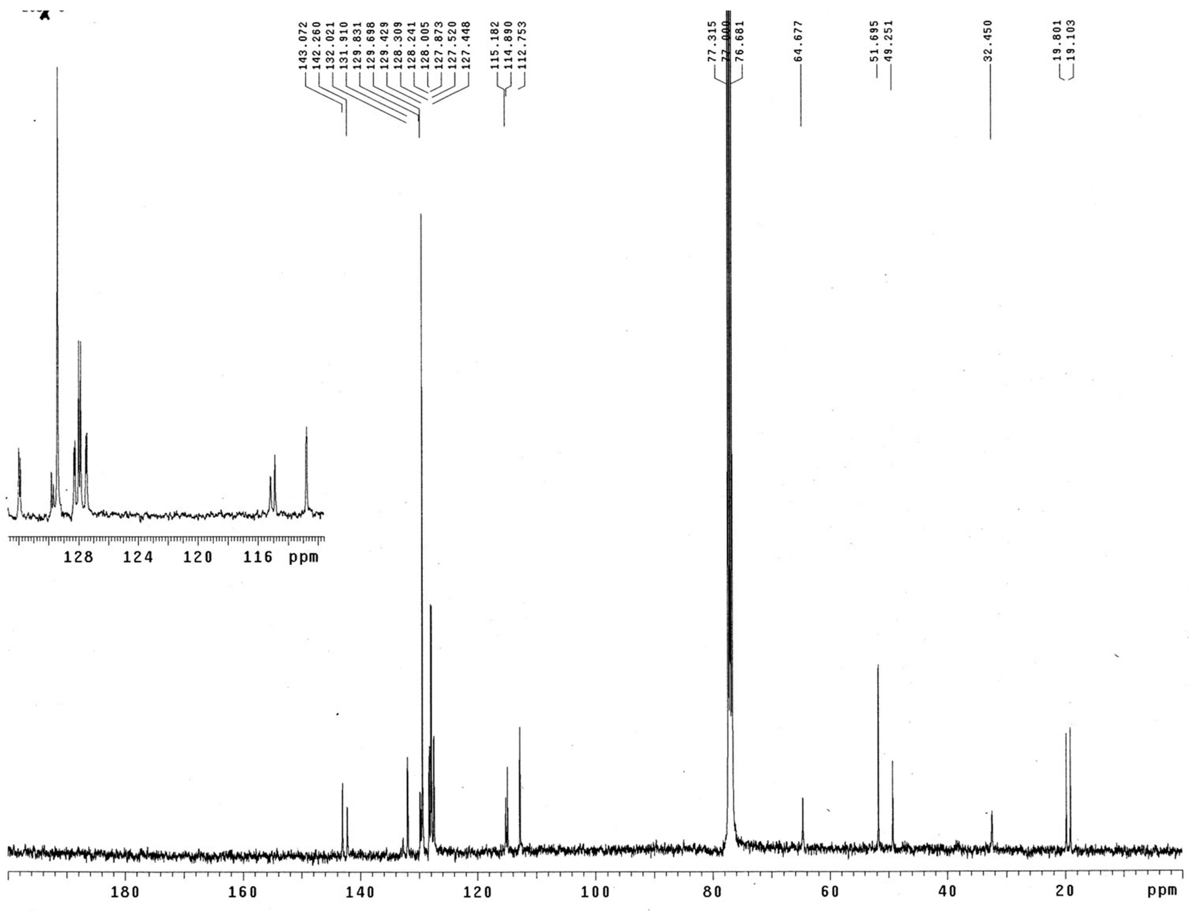

^{1}H NMR ($CDCl_3$, 400 MHz) spectrum of compound **5aa** (table 2)

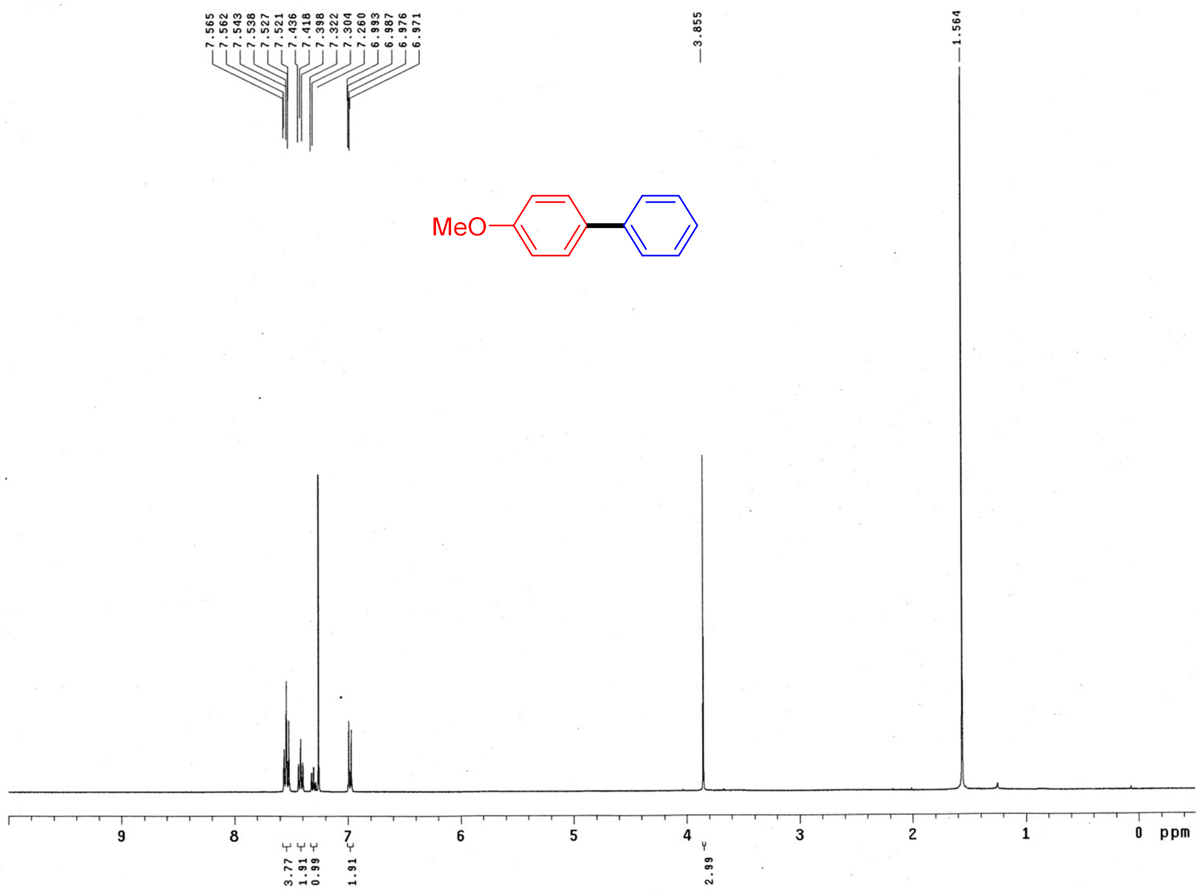

^{13}C NMR ($CDCl_3$, 100 MHz) spectrum of compound **5aa**

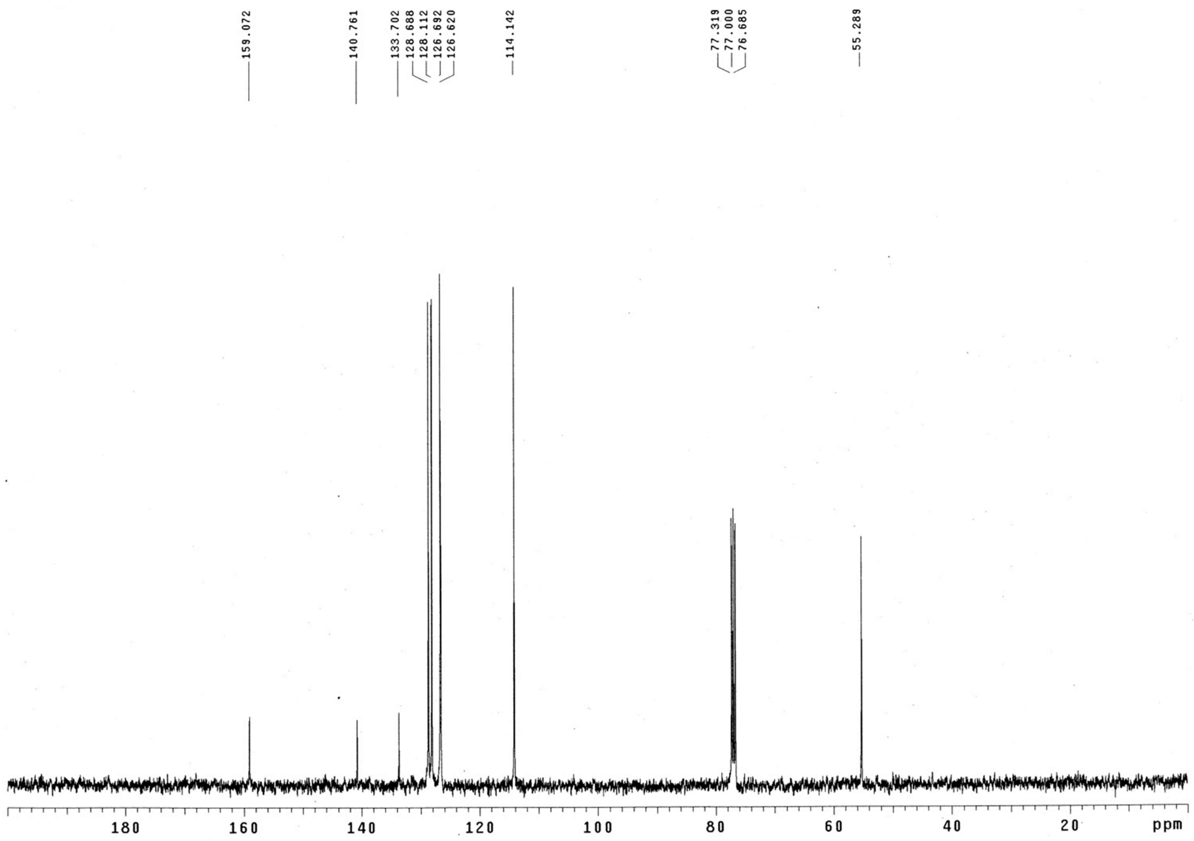

^{1}H NMR ($CDCl_3$, 400 MHz) spectrum of compound **5ba** (table 2)

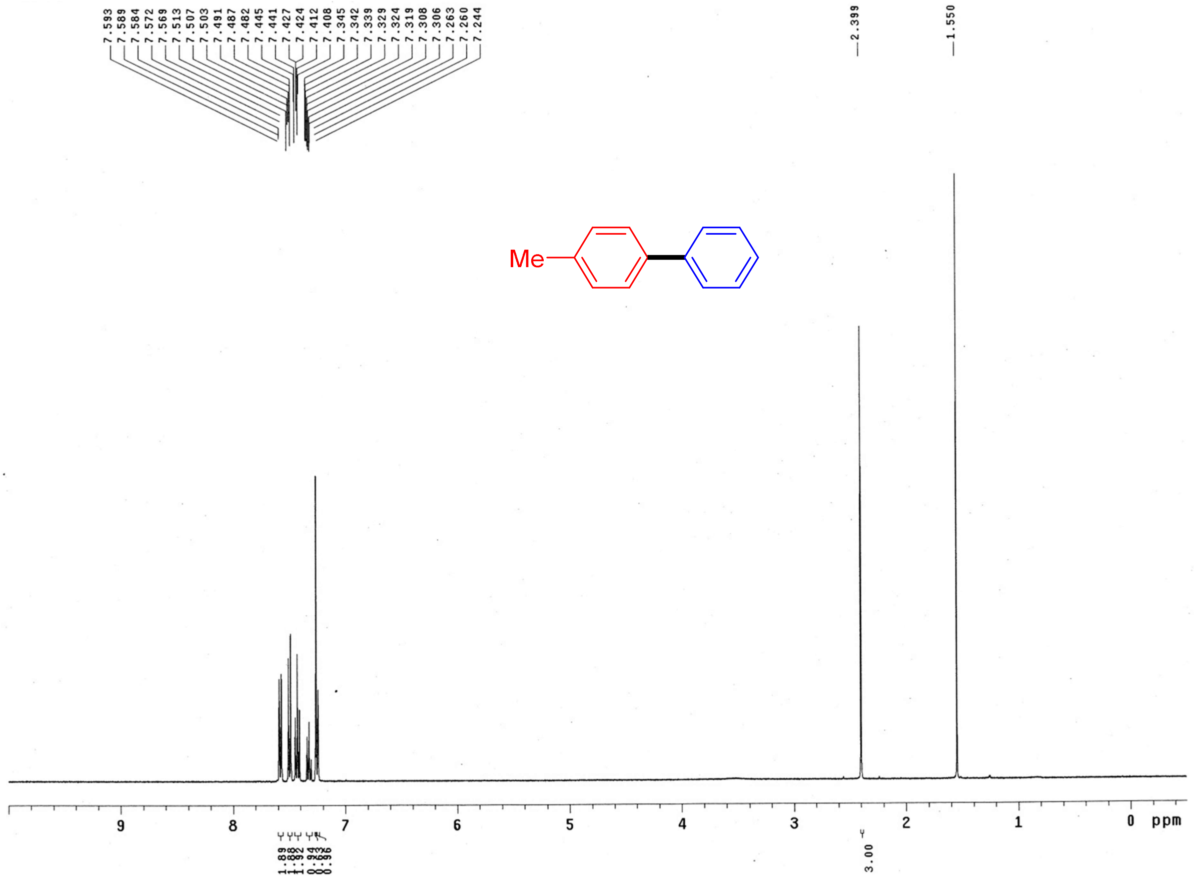

^{13}C NMR ($CDCl_3$, 100 MHz) spectrum of compound **5ba**

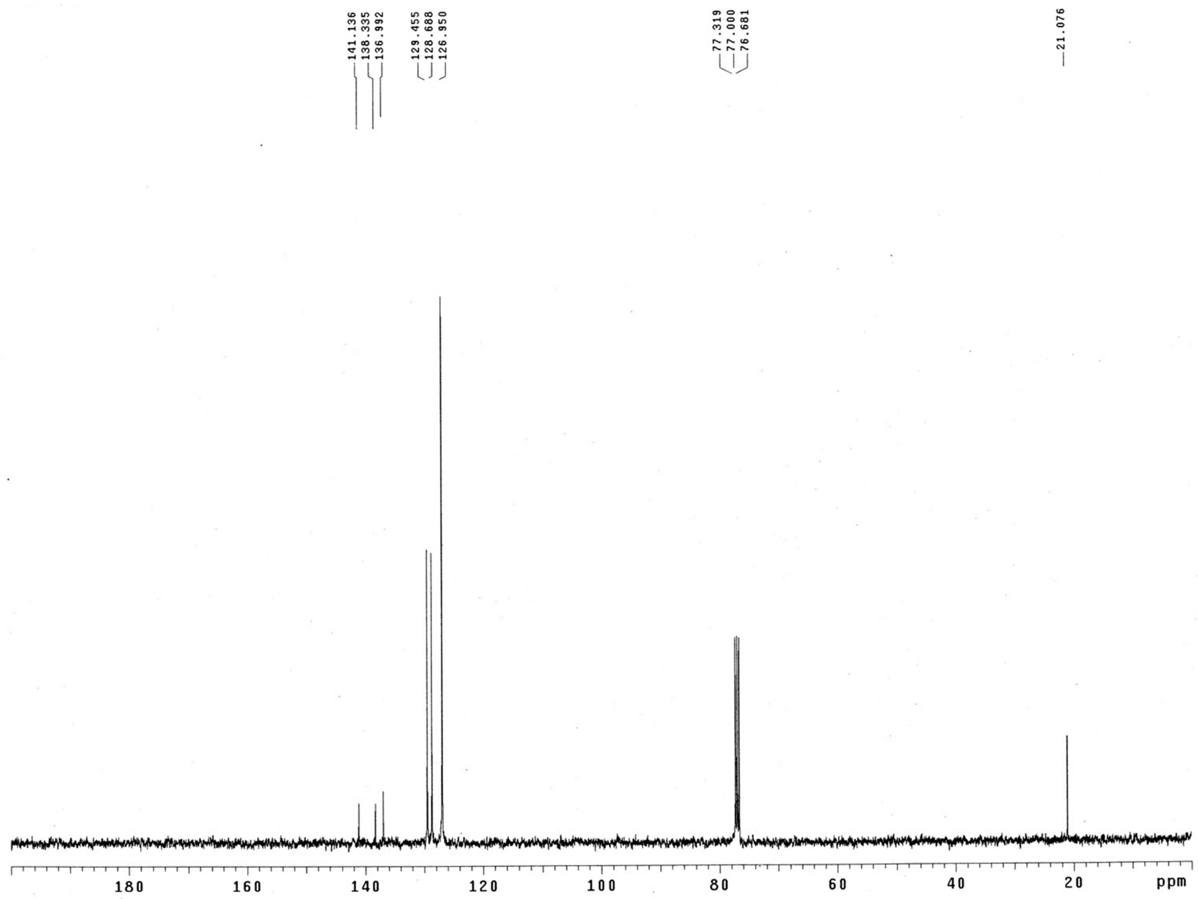

^{1}H NMR ($CDCl_3$, 400 MHz) spectrum of compound **5ca** (table 2)

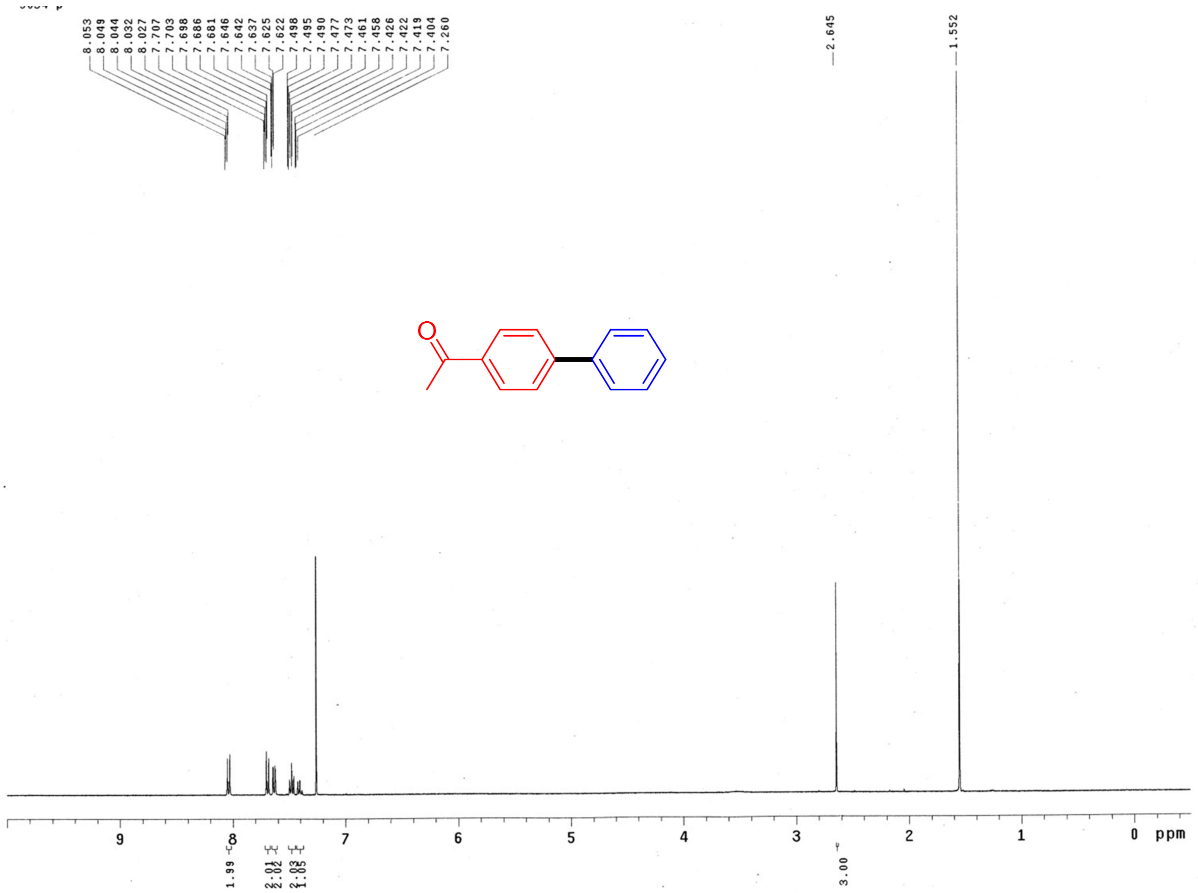

^{13}C NMR ($CDCl_3$, 100 MHz) spectrum of compound **5ca**

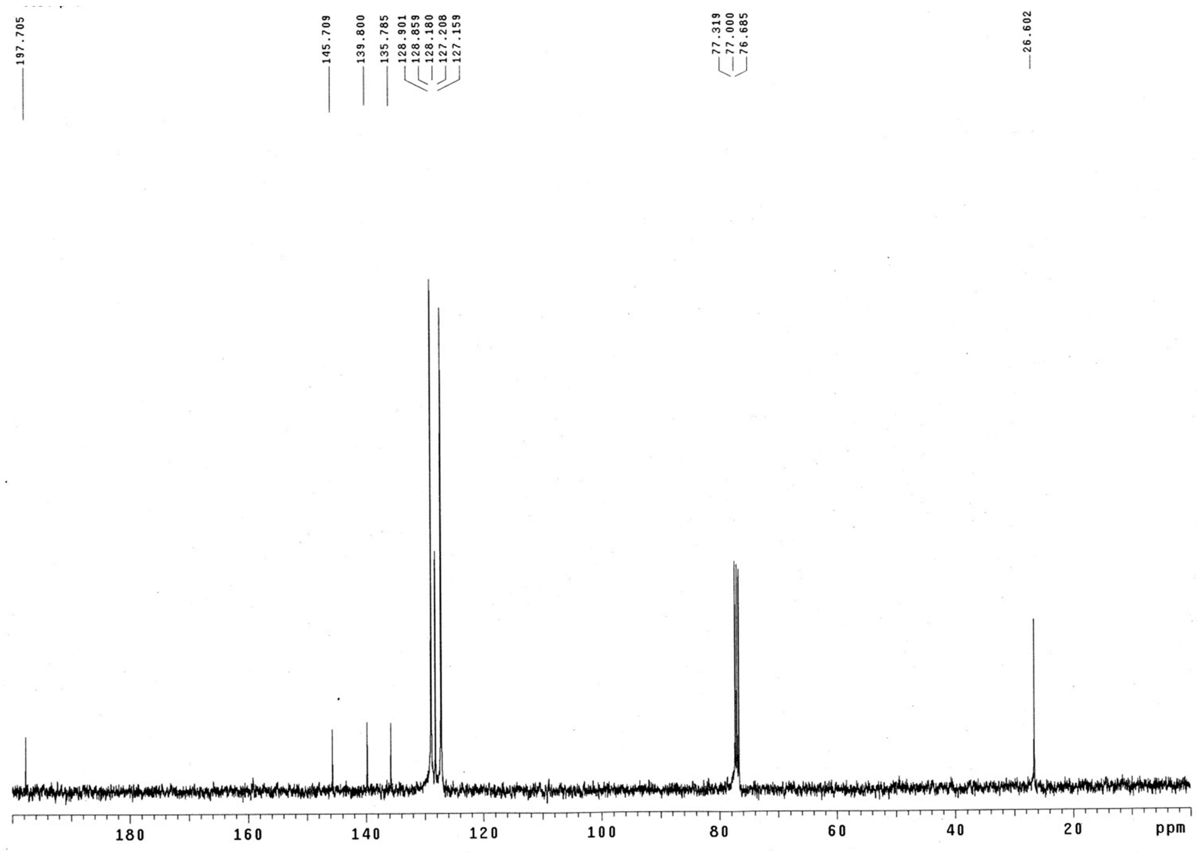

^{1}H NMR ($CDCl_3$, 400 MHz) spectrum of compound **5da** (table 2)

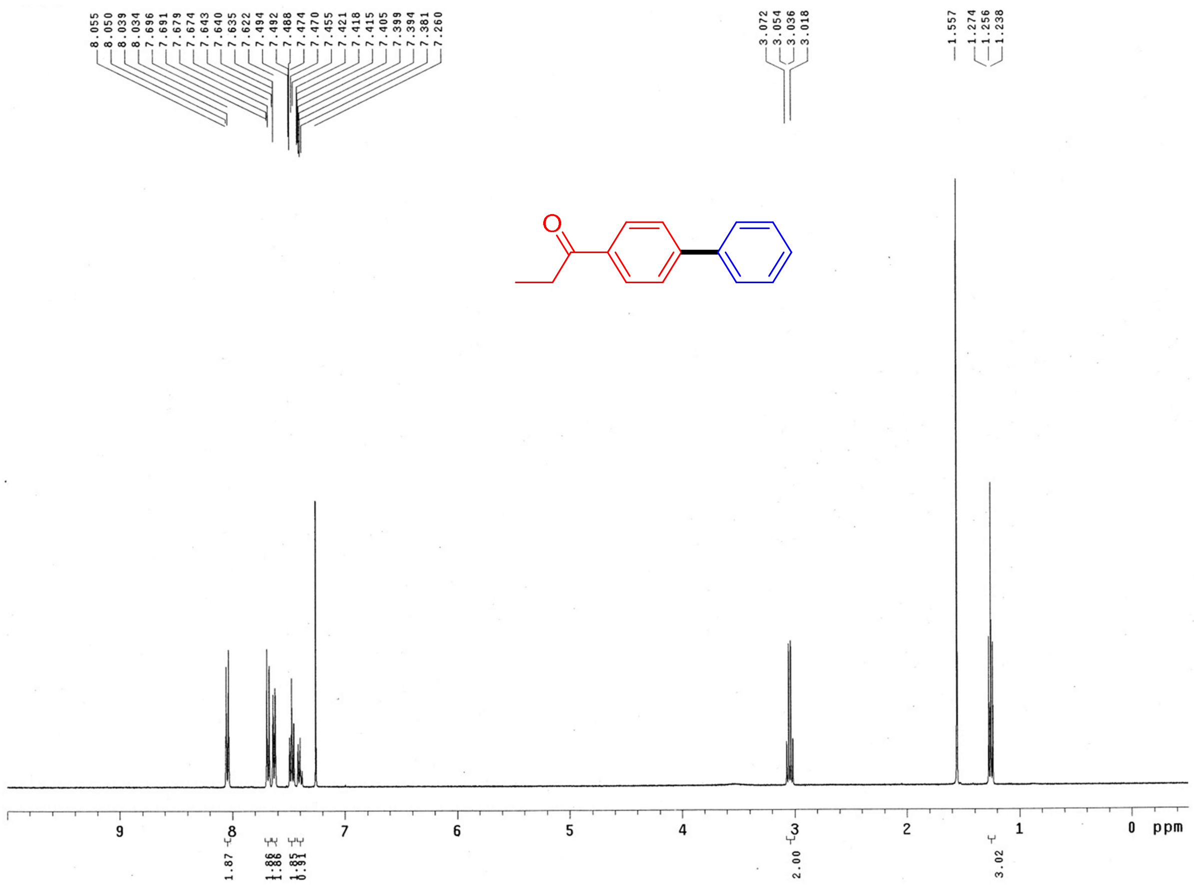

^{13}C NMR ($CDCl_3$, 100 MHz) spectrum of compound **5da**

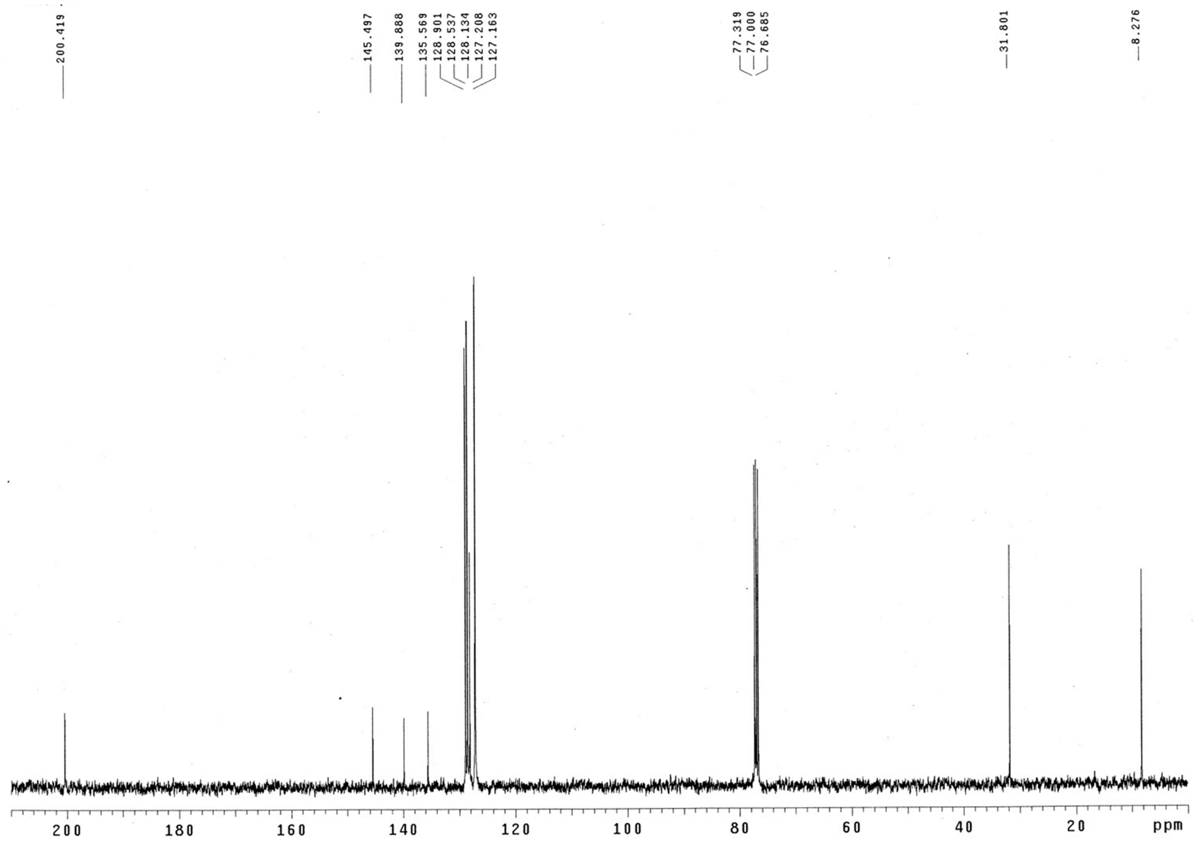

^{1}H NMR ($CDCl_3$, 400 MHz) spectrum of compound **5ea** (table 2)

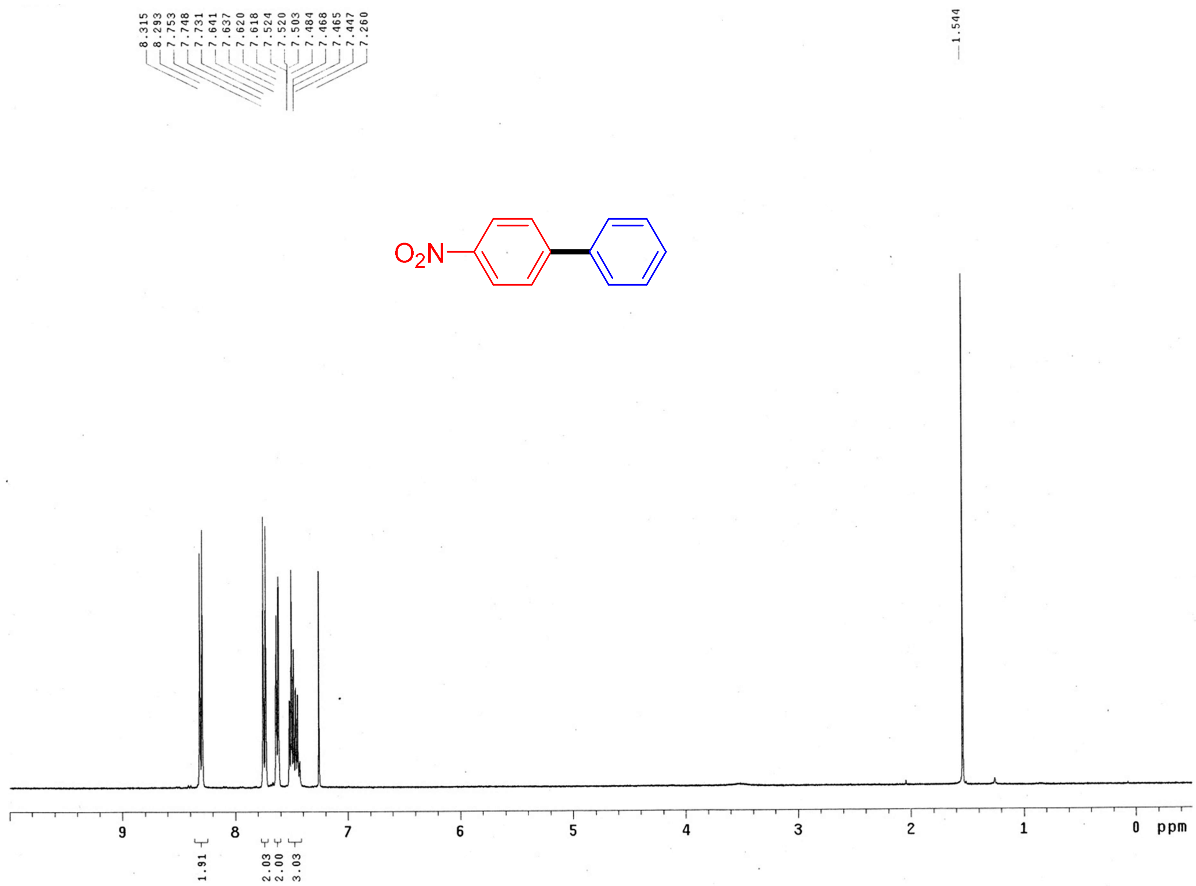

^{13}C NMR ($CDCl_3$, 100 MHz) spectrum of compound **5ea**

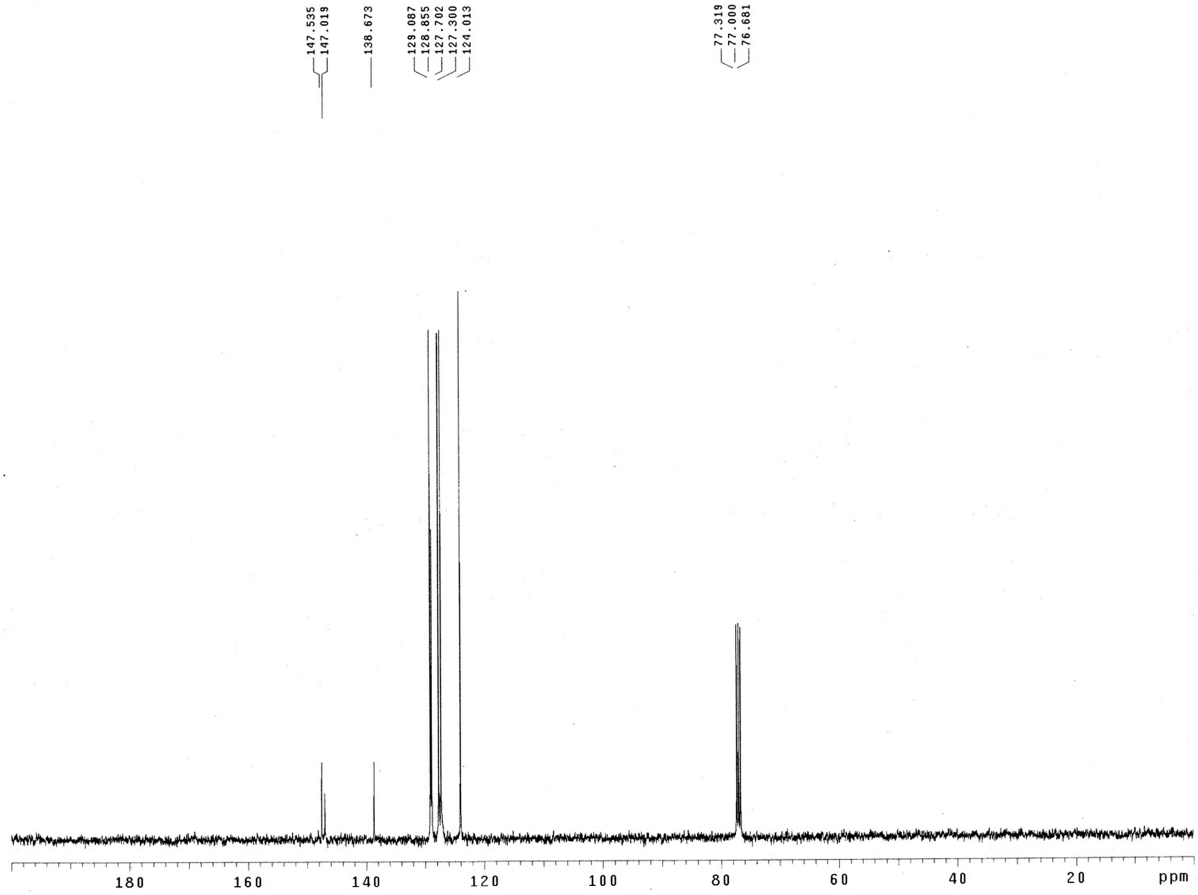

^{1}H NMR ($CDCl_3$, 400 MHz) spectrum of compound **5fa** (table 2)

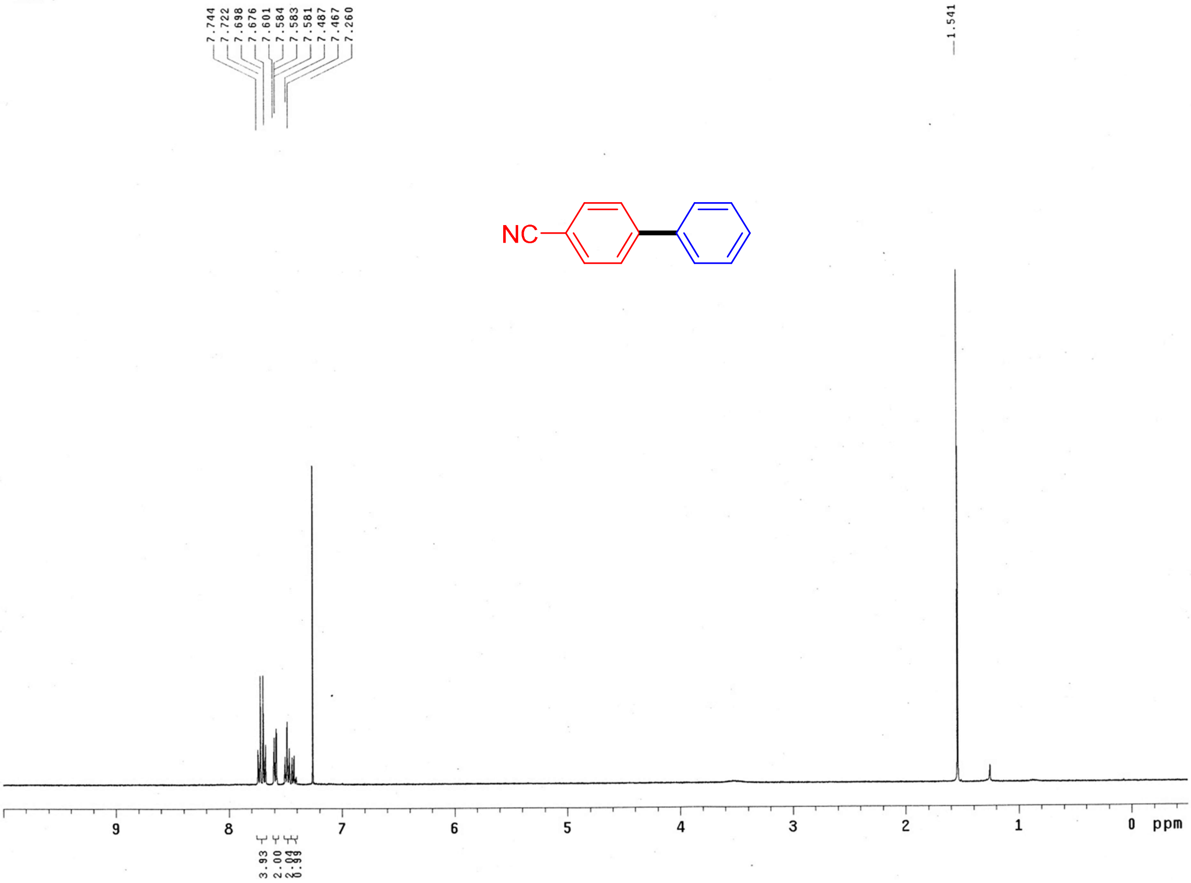

^{13}C NMR ($CDCl_3$, 100 MHz) spectrum of compound **5fa**

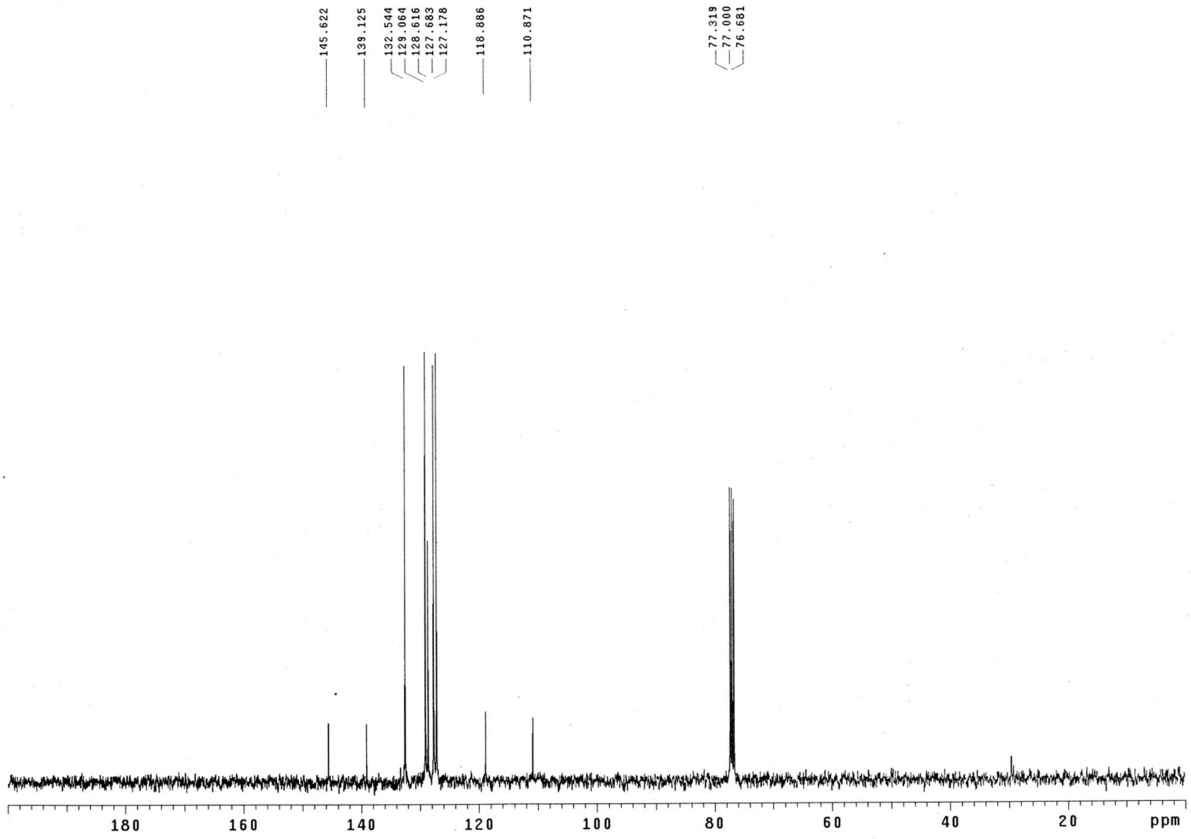

^{1}H NMR ($CDCl_3$, 400 MHz) spectrum of compound **5ga** (table 2)

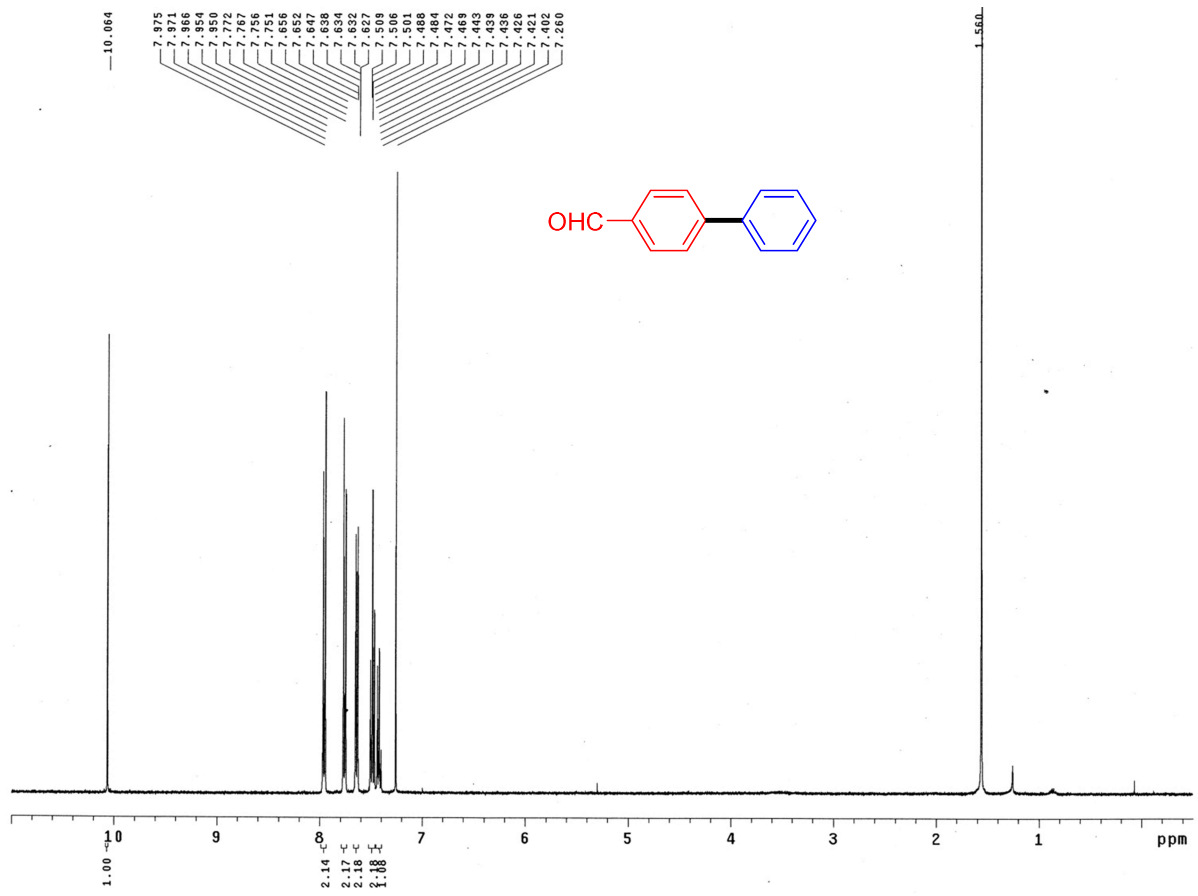

^{13}C NMR ($CDCl_3$, 100 MHz) spectrum of compound **5ga**

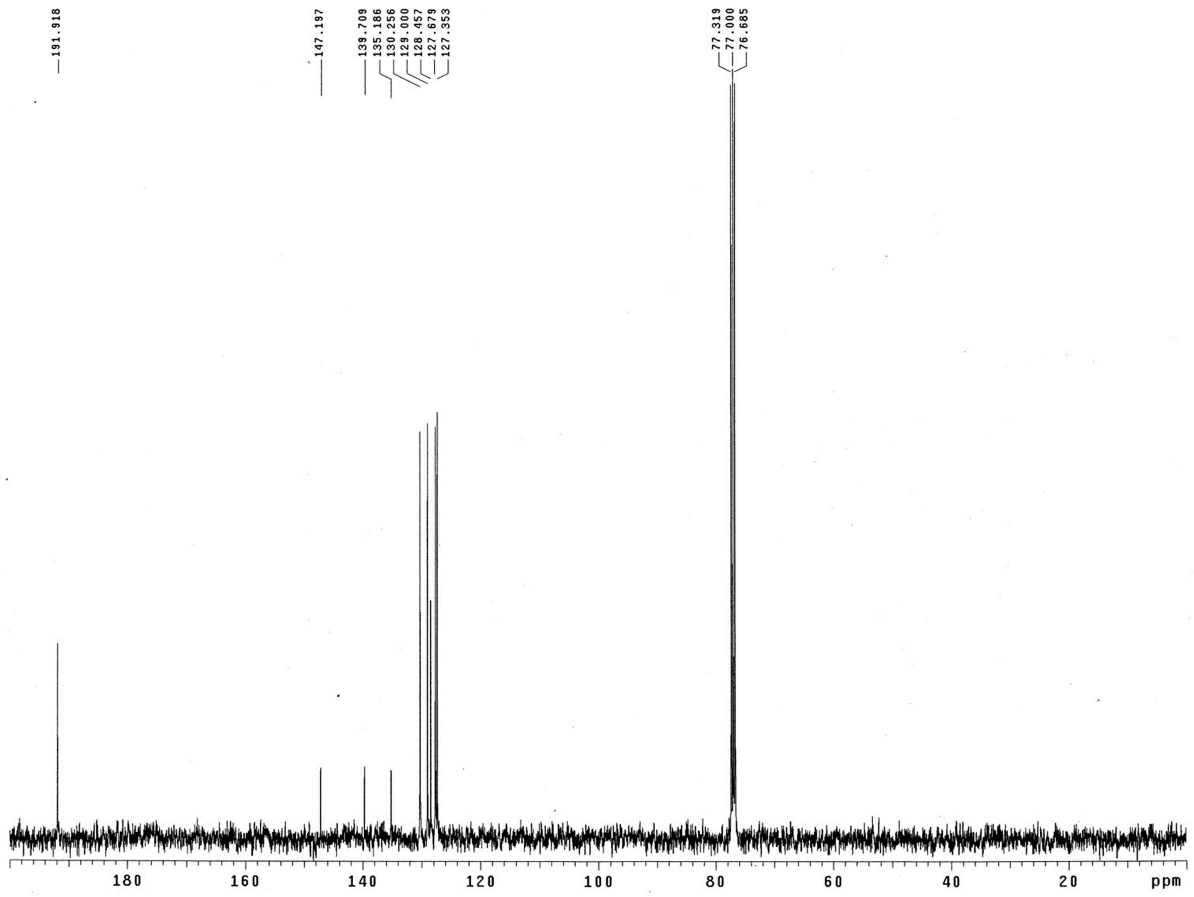

^{1}H NMR ($CDCl_3$, 400 MHz) spectrum of compound **5ha** (table 2)

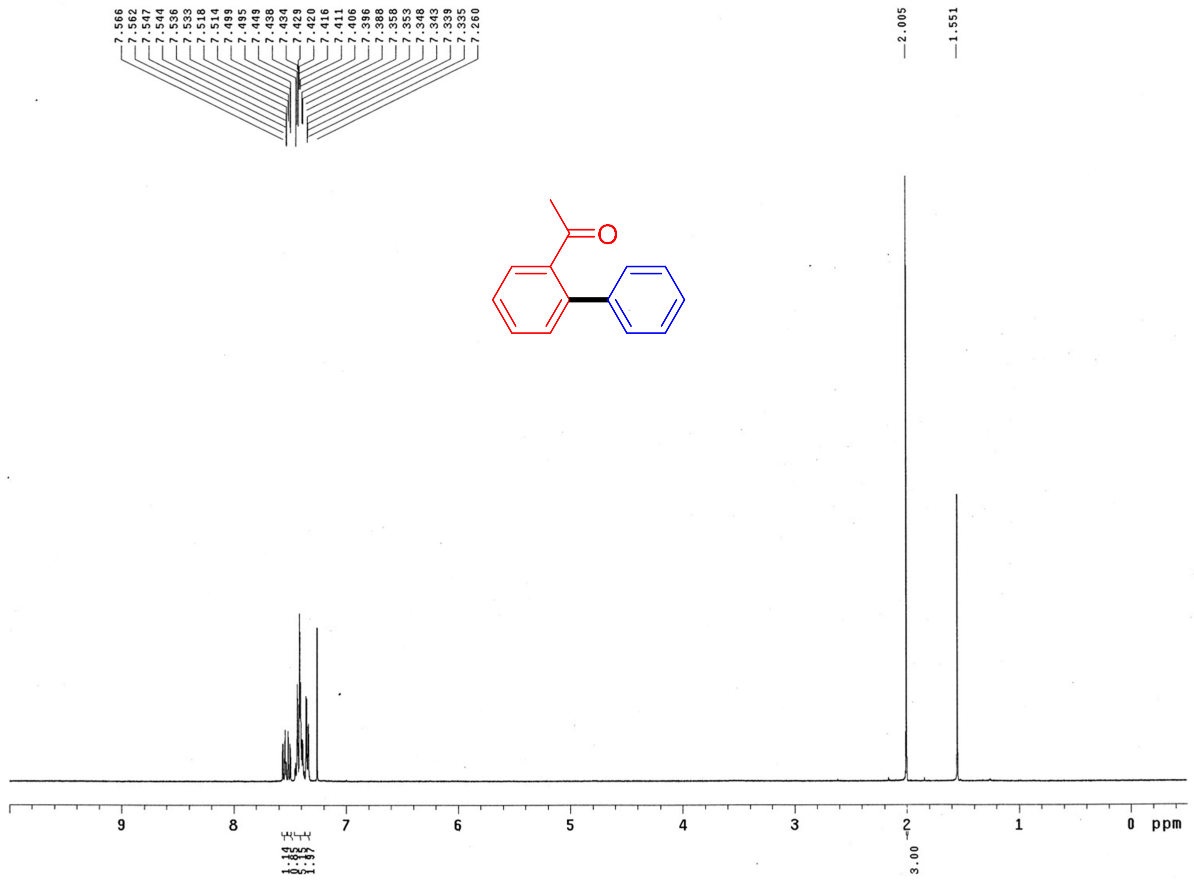

^{13}C NMR ($CDCl_3$, 100 MHz) spectrum of compound **5ha**

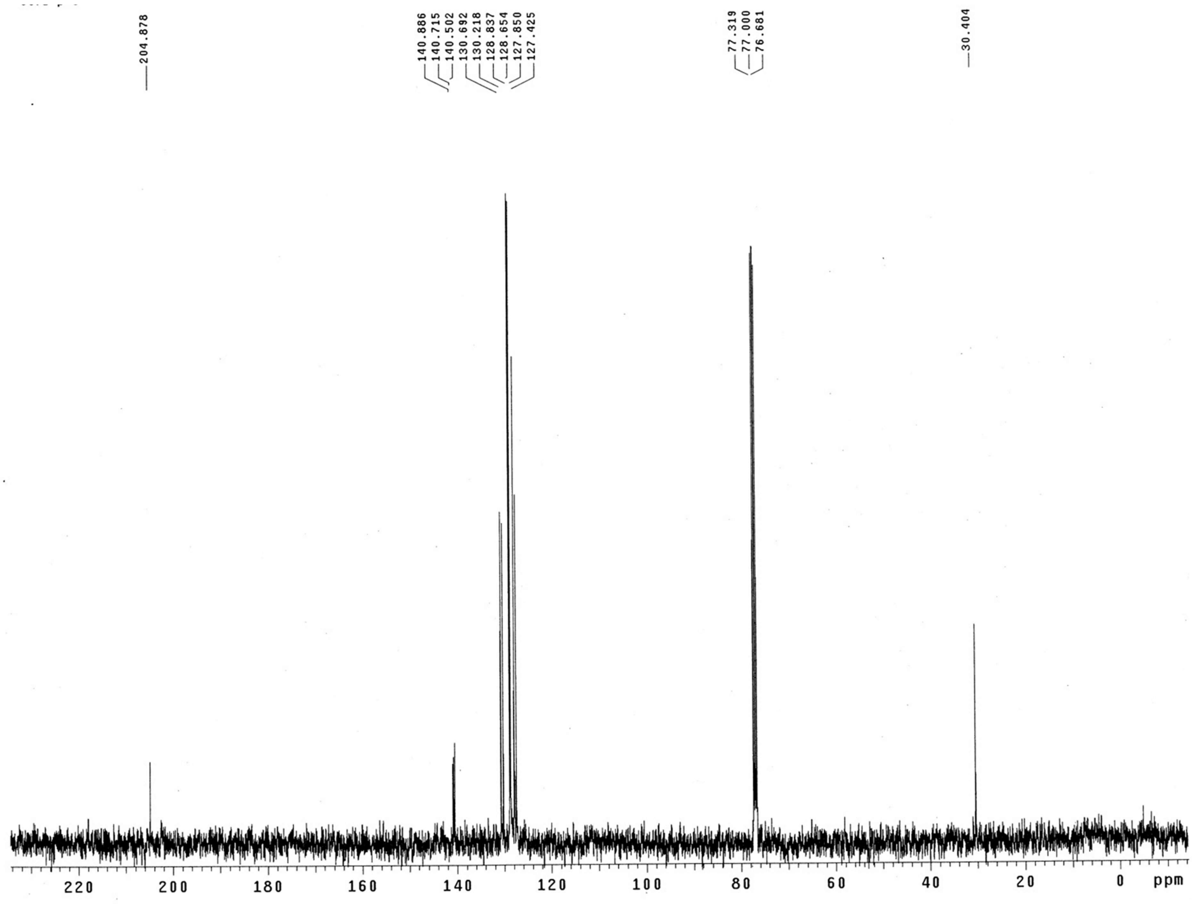

^{1}H NMR ($CDCl_3$, 400 MHz) spectrum of compound **5ia** (table 2)

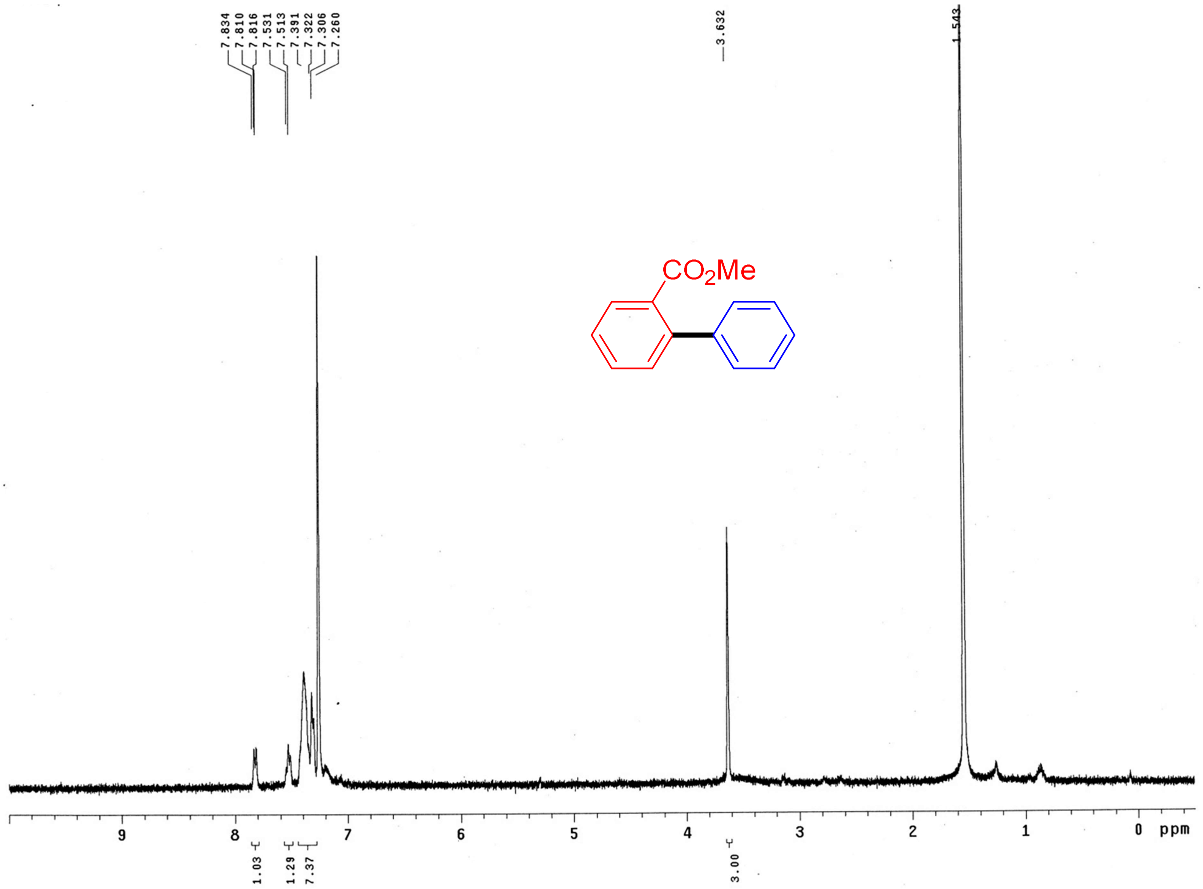

^{13}C NMR ($CDCl_3$, 100 MHz) spectrum of compound **5ia**

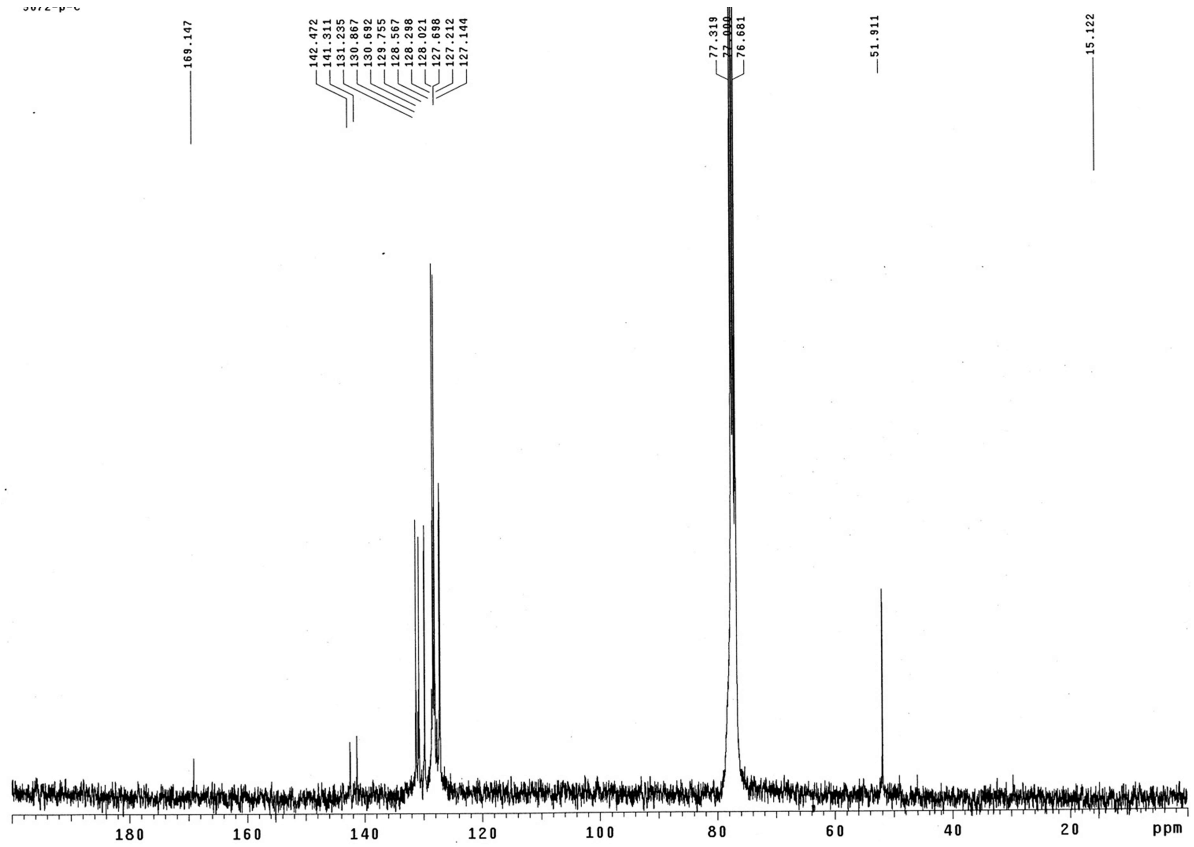

^{1}H NMR ($CDCl_3$, 400 MHz) spectrum of compound **5ja** (table 2)

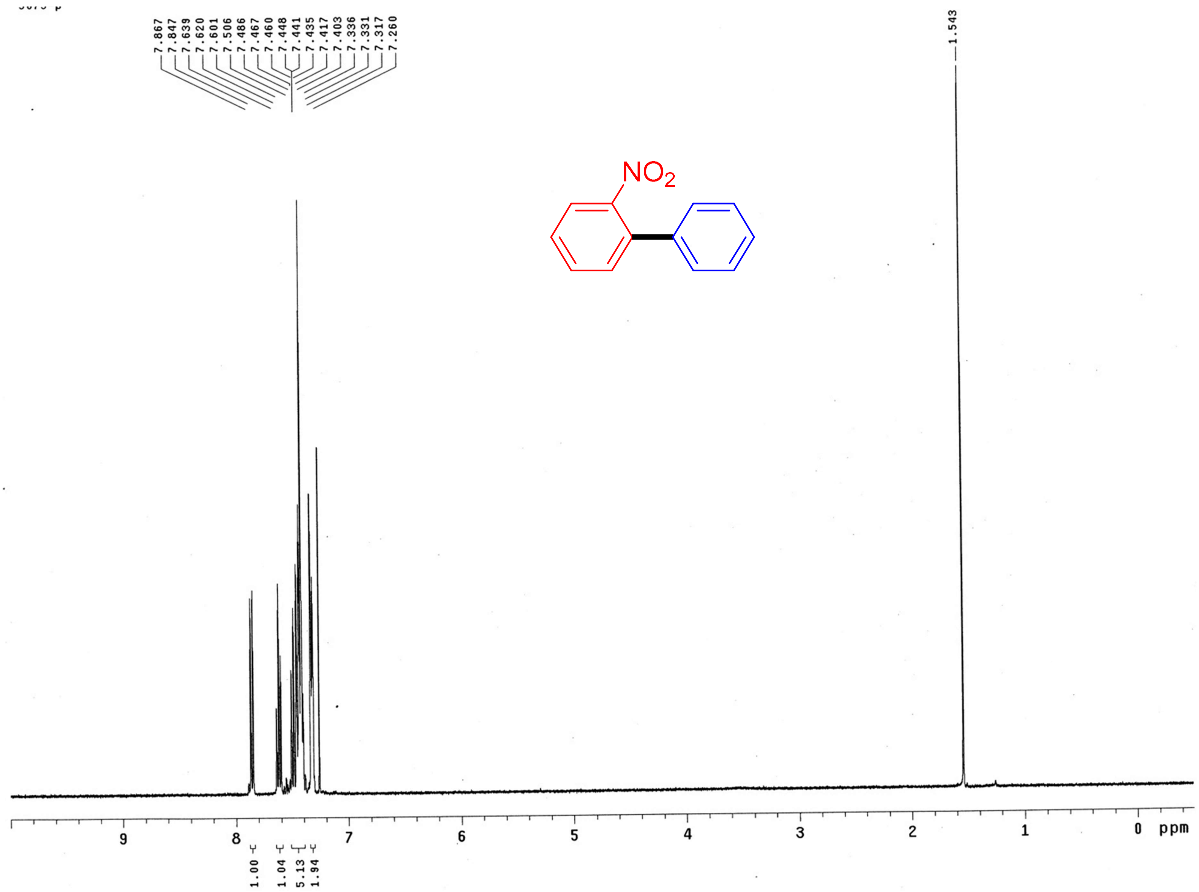

^{13}C NMR ($CDCl_3$, 100 MHz) spectrum of compound **5ja**

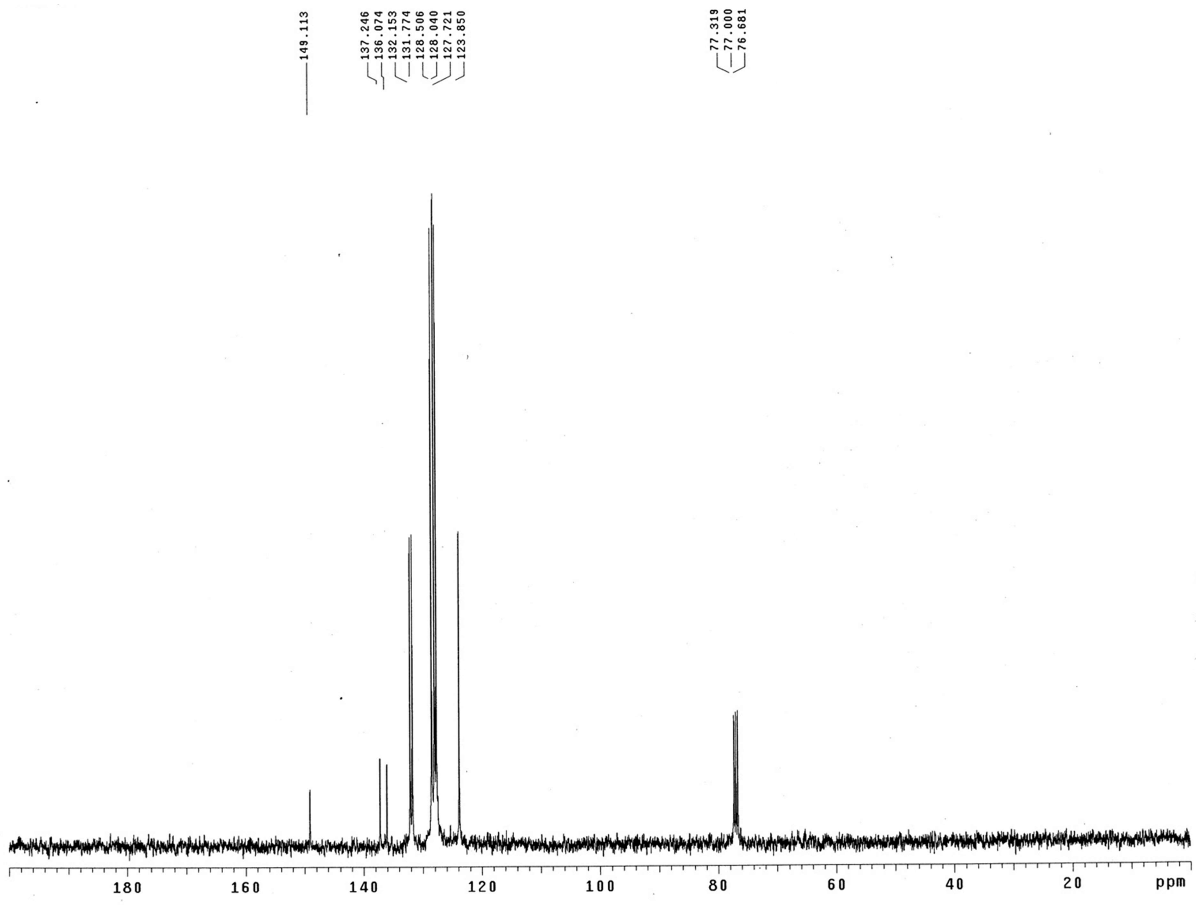

^{1}H NMR ($CDCl_3$, 400 MHz) spectrum of compound **5ka** (table 2)

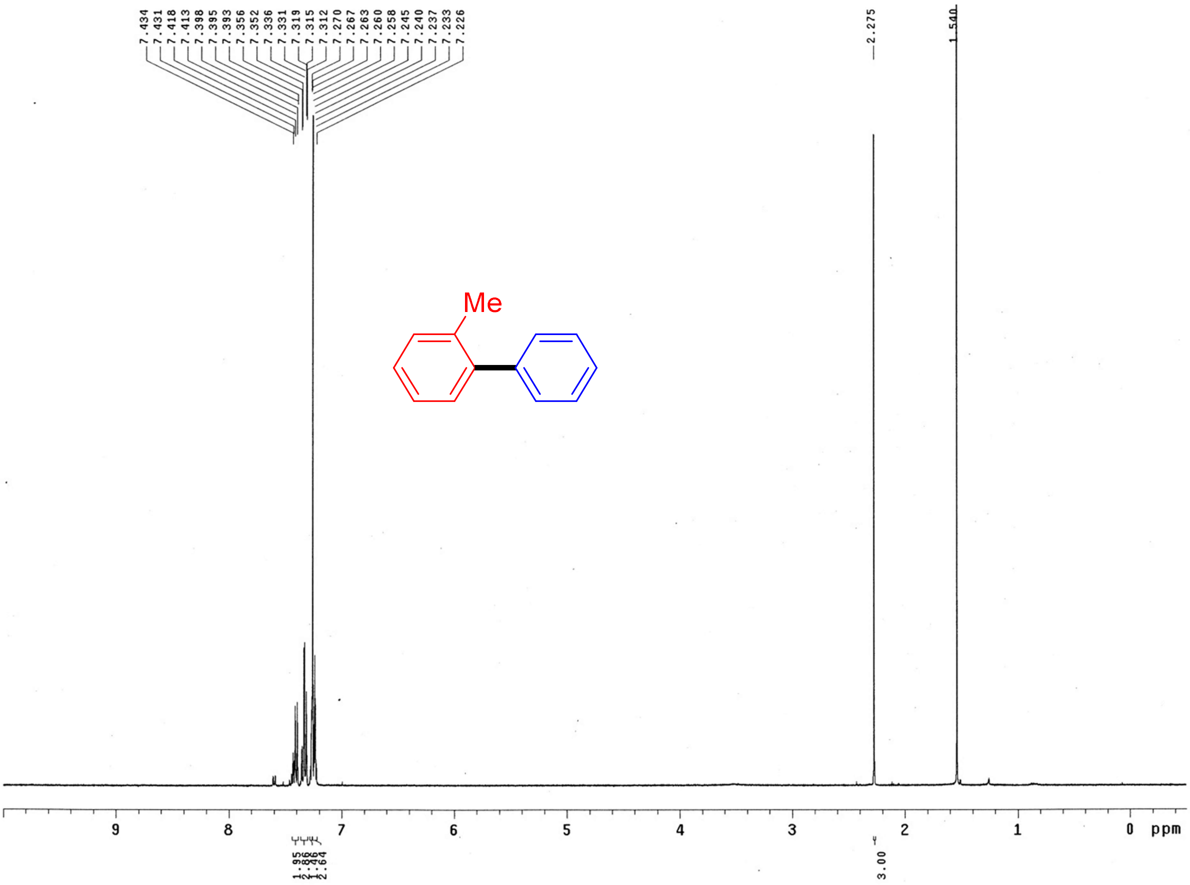

^{13}C NMR ($CDCl_3$, 100 MHz) spectrum of compound **5ka**

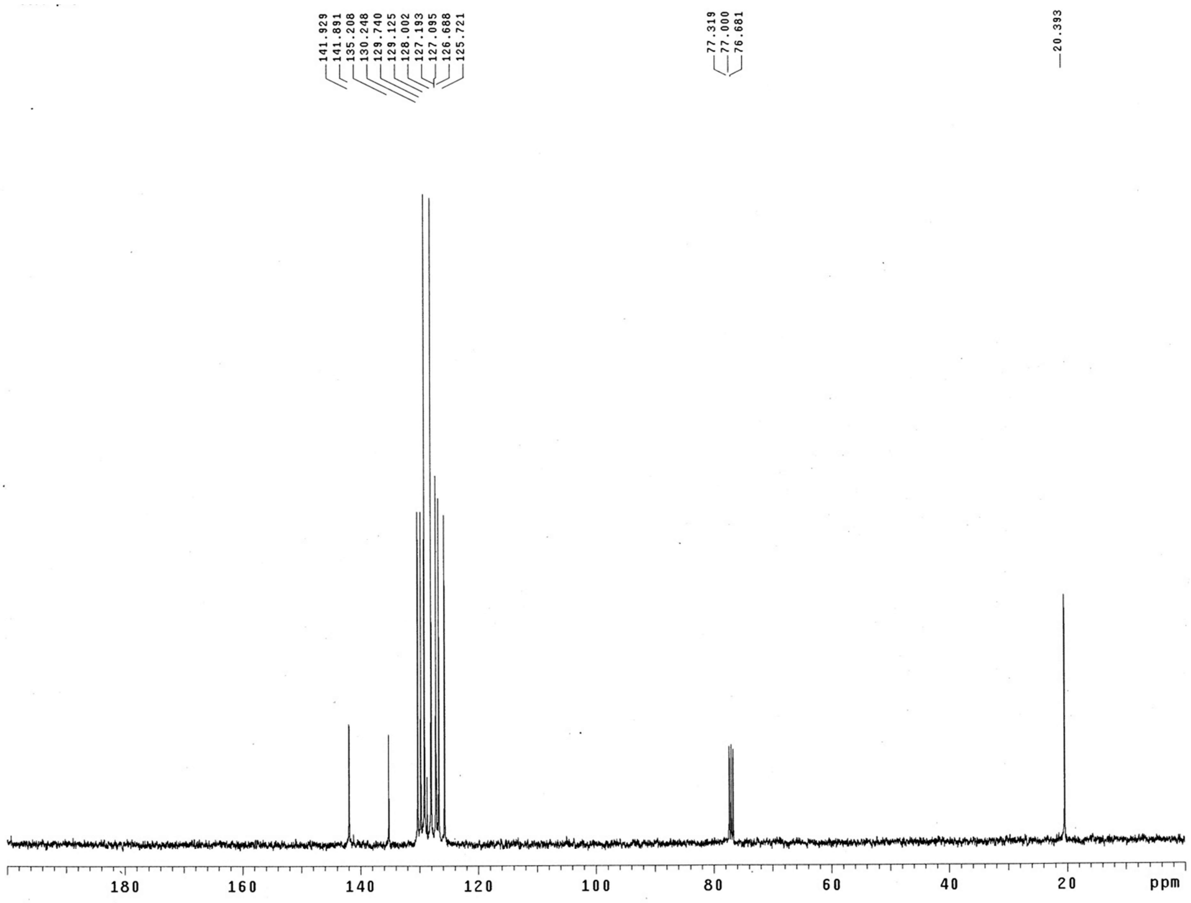

^{1}H NMR ($CDCl_3$, 400 MHz) spectrum of compound **5ma** (table 3)

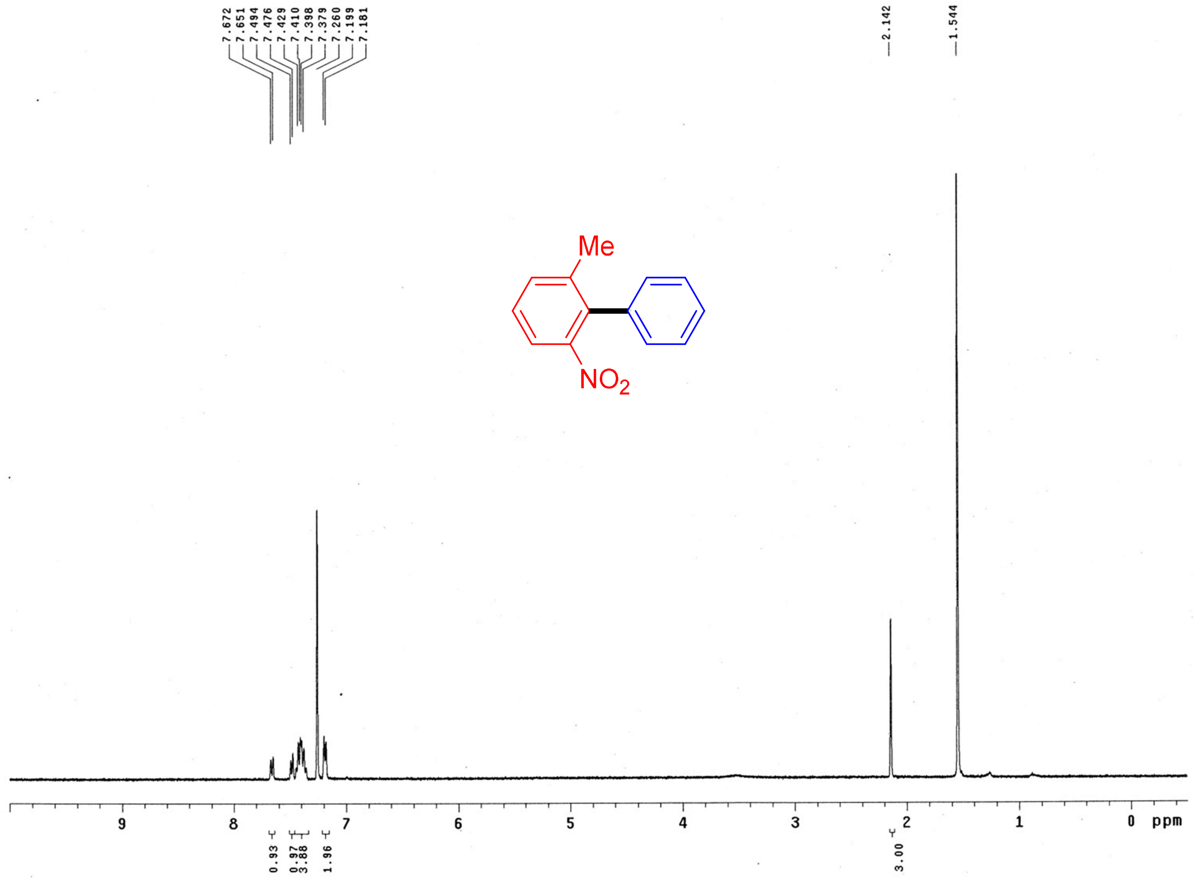

^{13}C NMR ($CDCl_3$, 100 MHz) spectrum of compound **5ma**

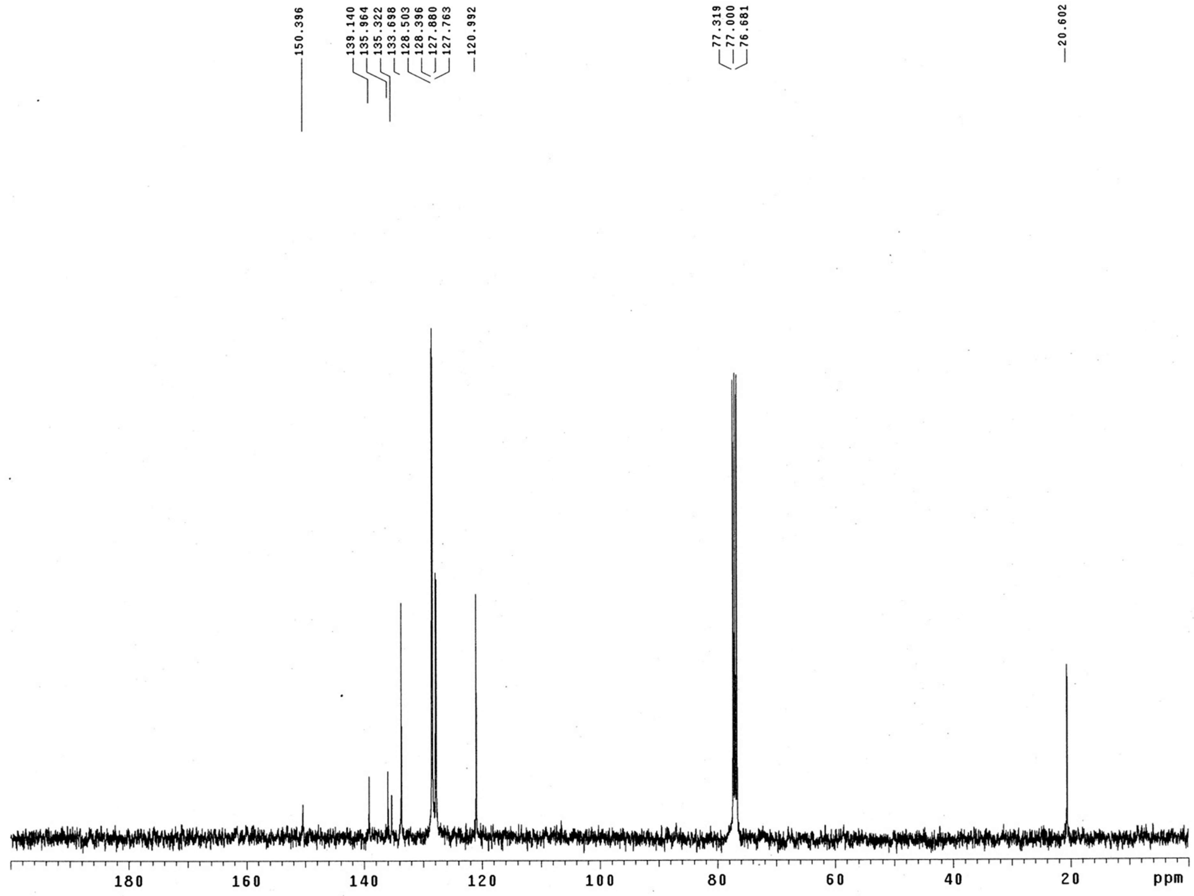

^{1}H NMR ($CDCl_3$, 400 MHz) spectrum of compound **5na** (table 3)

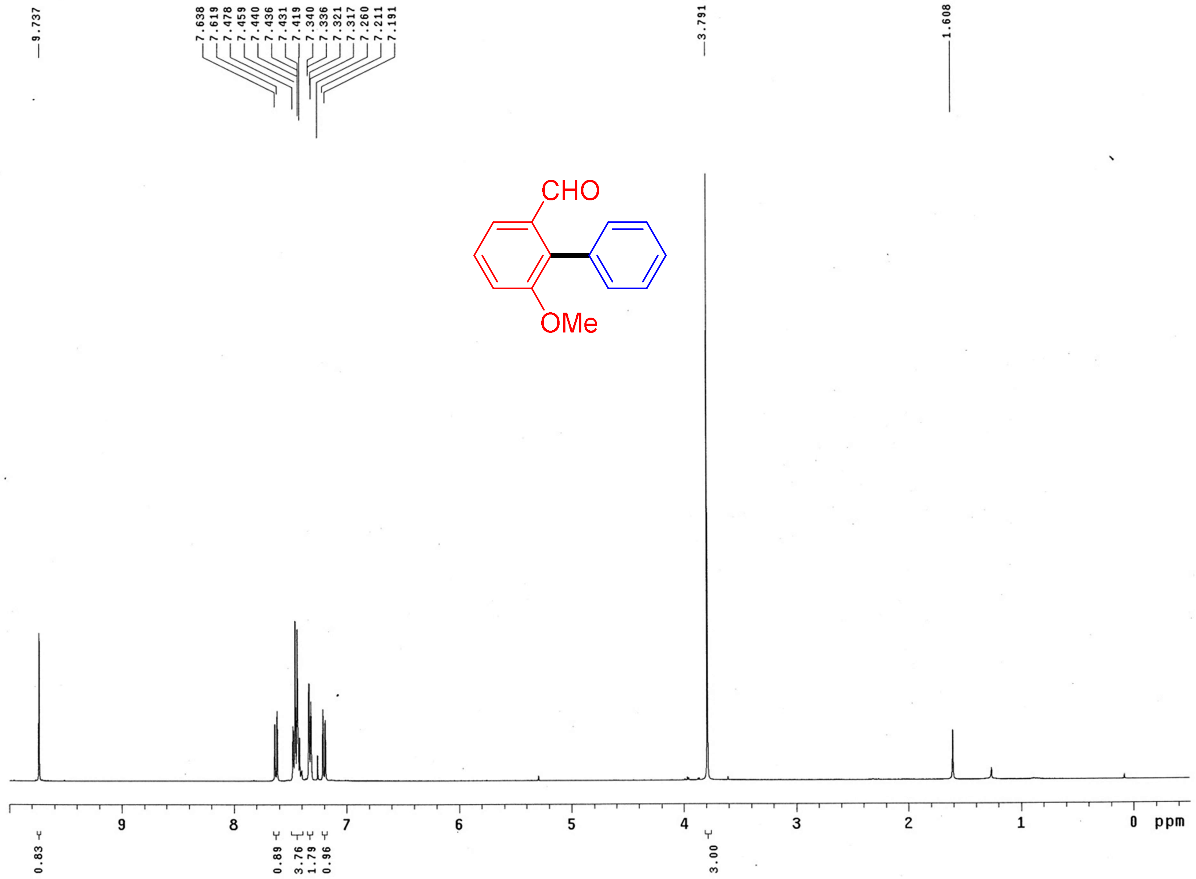

^{13}C NMR ($CDCl_3$, 100 MHz) spectrum of compound **5na**

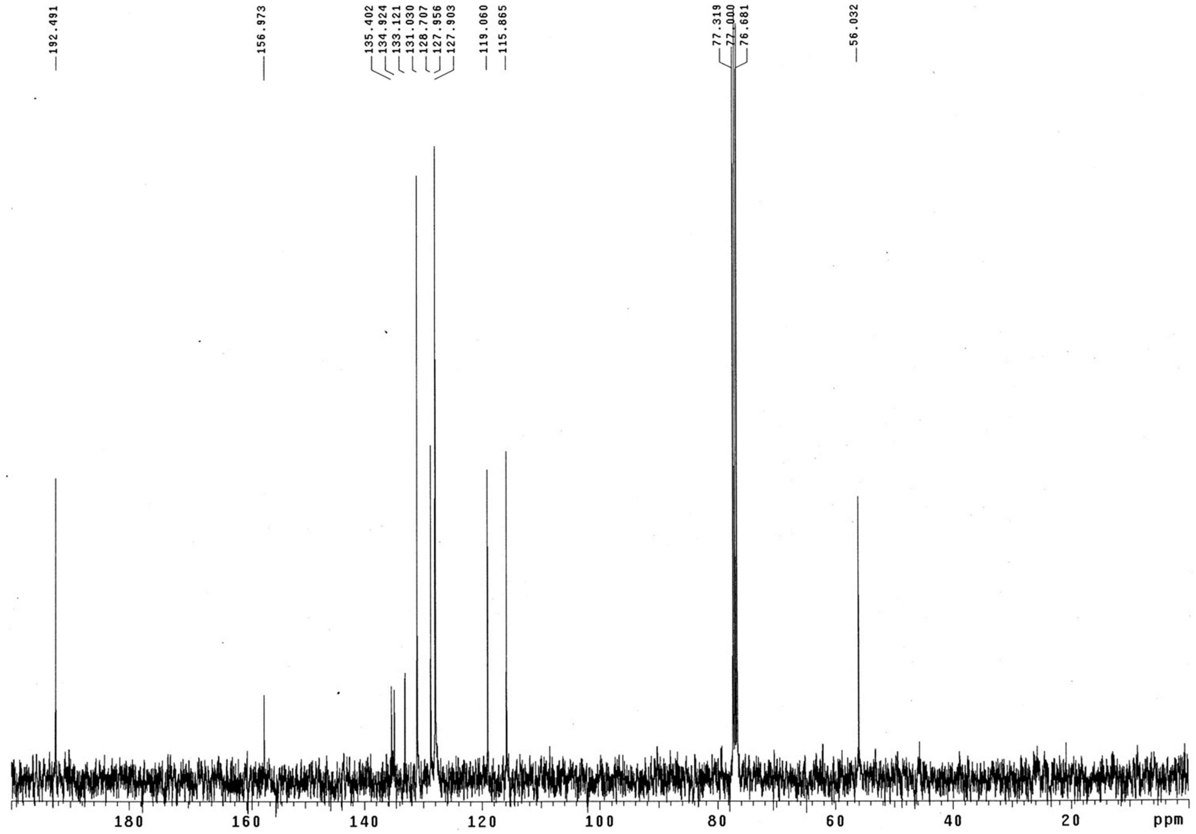

^{1}H NMR ($CDCl_3$, 400 MHz) spectrum of compound **5oa** (table 3)

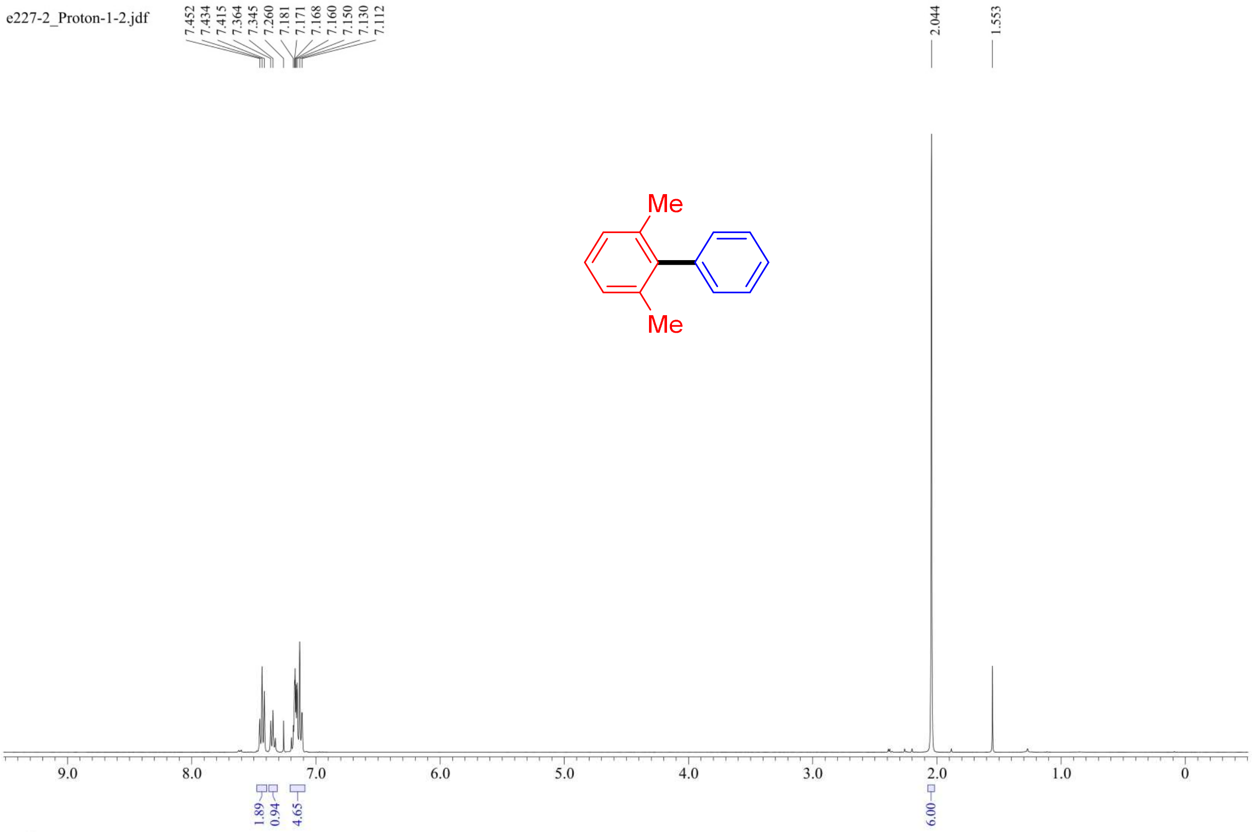

^{13}C NMR ($CDCl_3$, 100 MHz) spectrum of compound **5oa**

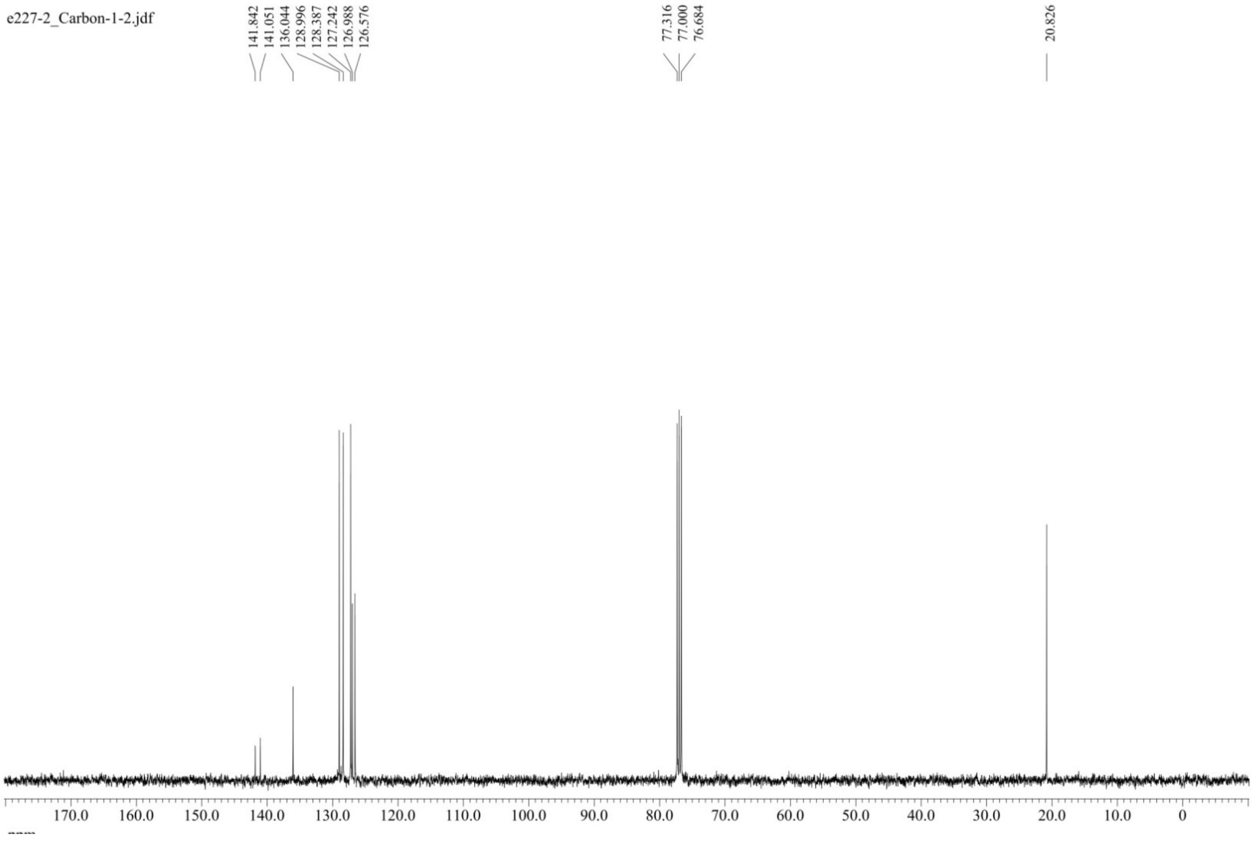

^{1}H NMR ($CDCl_3$, 400 MHz) spectrum of compound **5hb** (table 3)

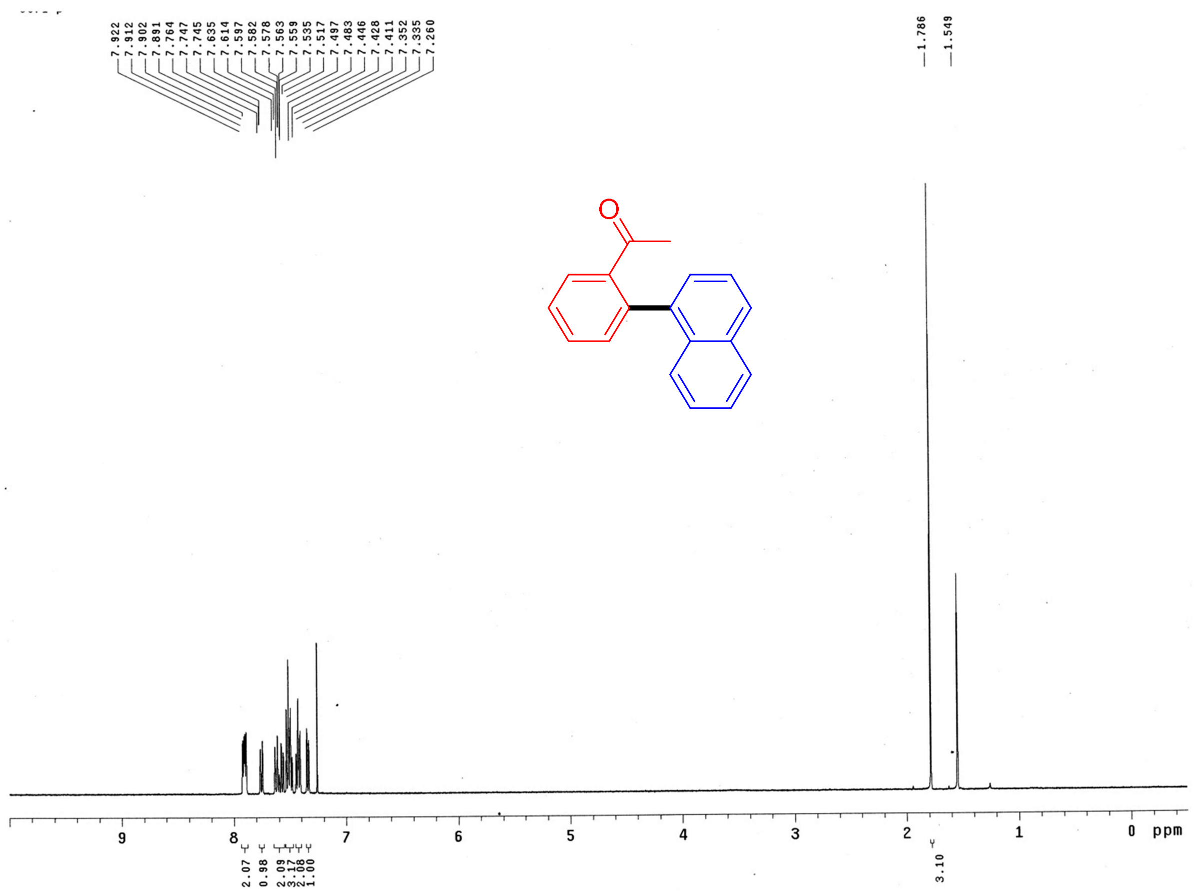

^{13}C NMR ($CDCl_3$, 100 MHz) spectrum of compound **5hb**

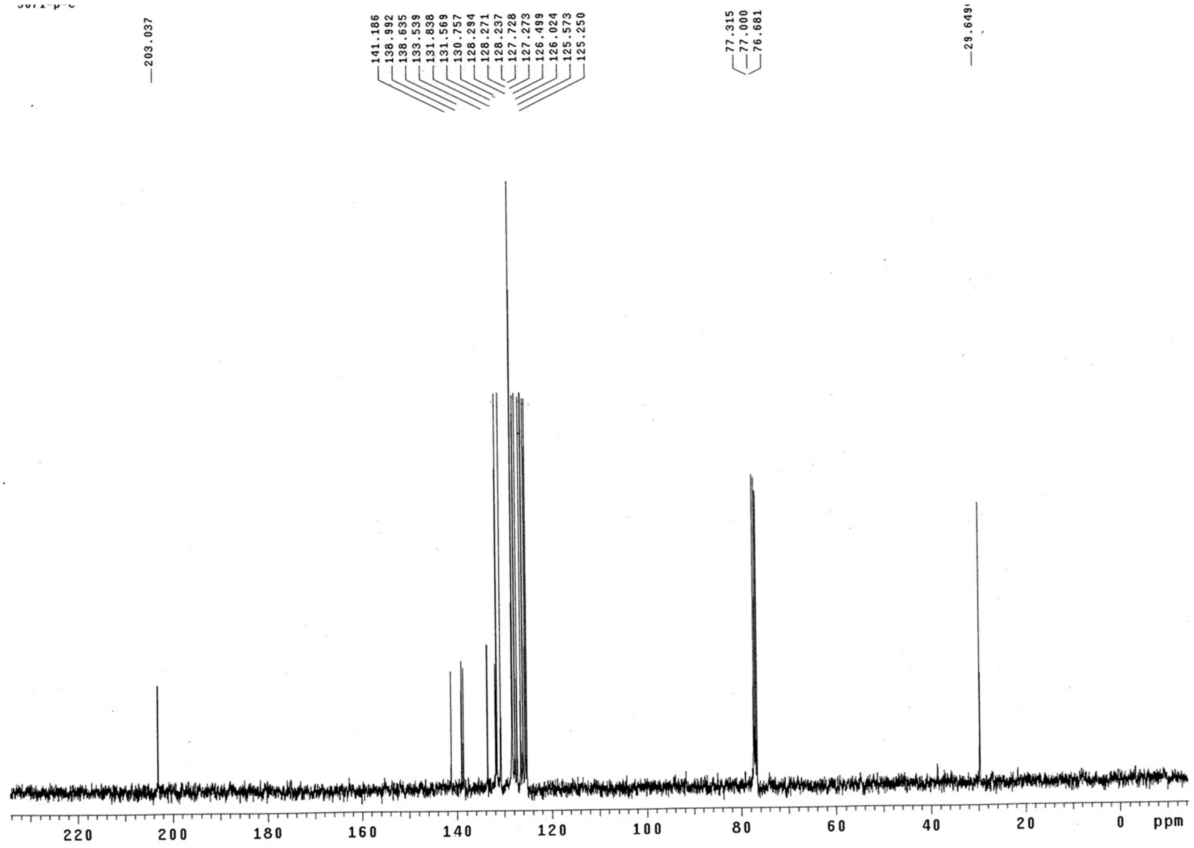

^{1}H NMR ($CDCl_3$, 400 MHz) spectrum of compound **5ib** (table 3)

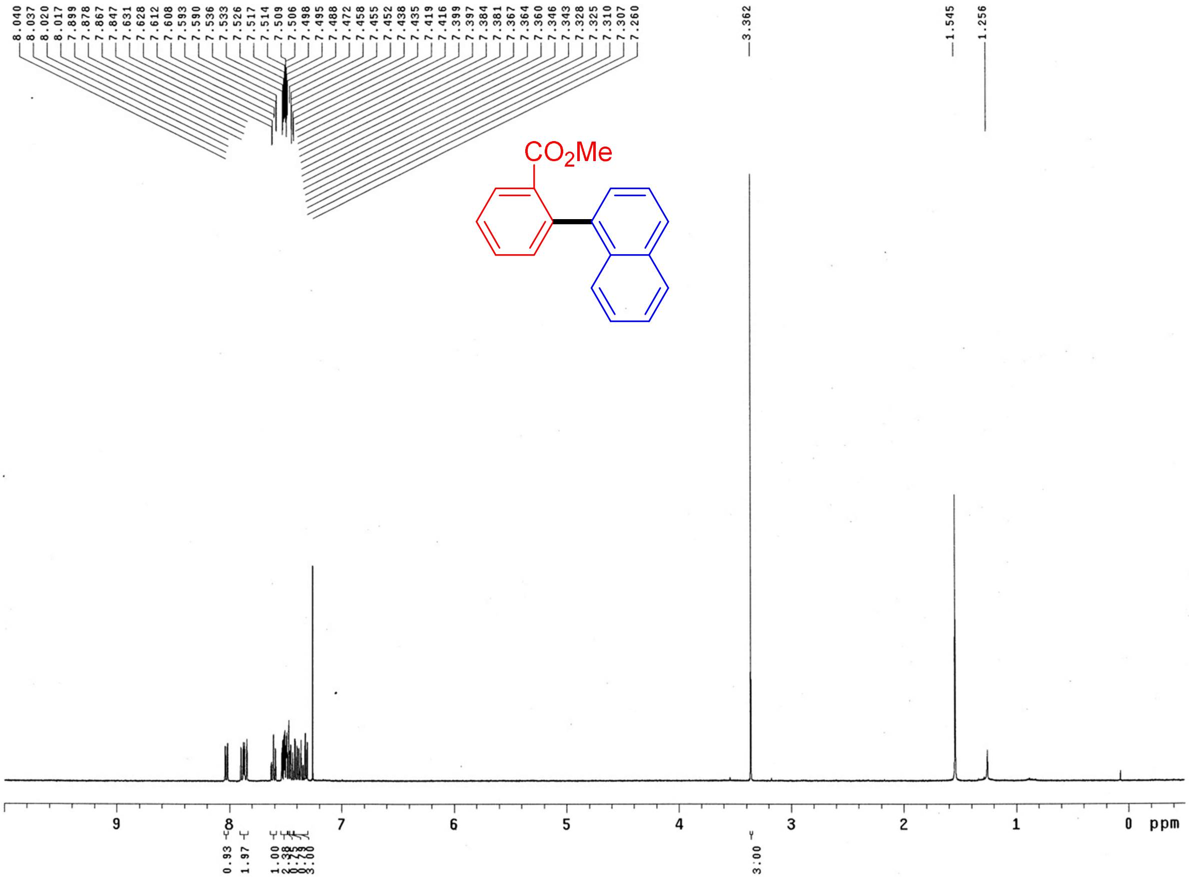

^{13}C NMR ($CDCl_3$, 100 MHz) spectrum of compound **5ib**

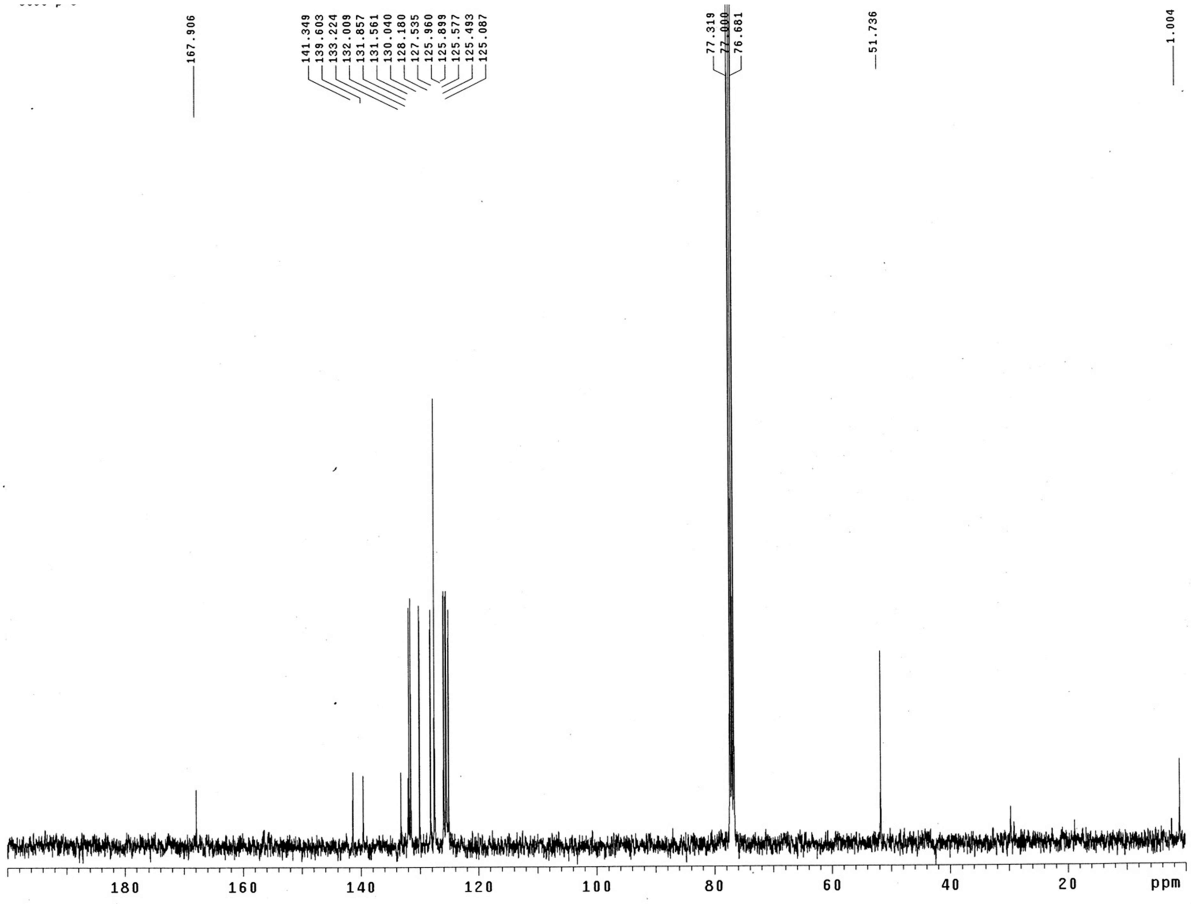

^{1}H NMR ($CDCl_3$, 400 MHz) spectrum of compound **5pb** (table 3)

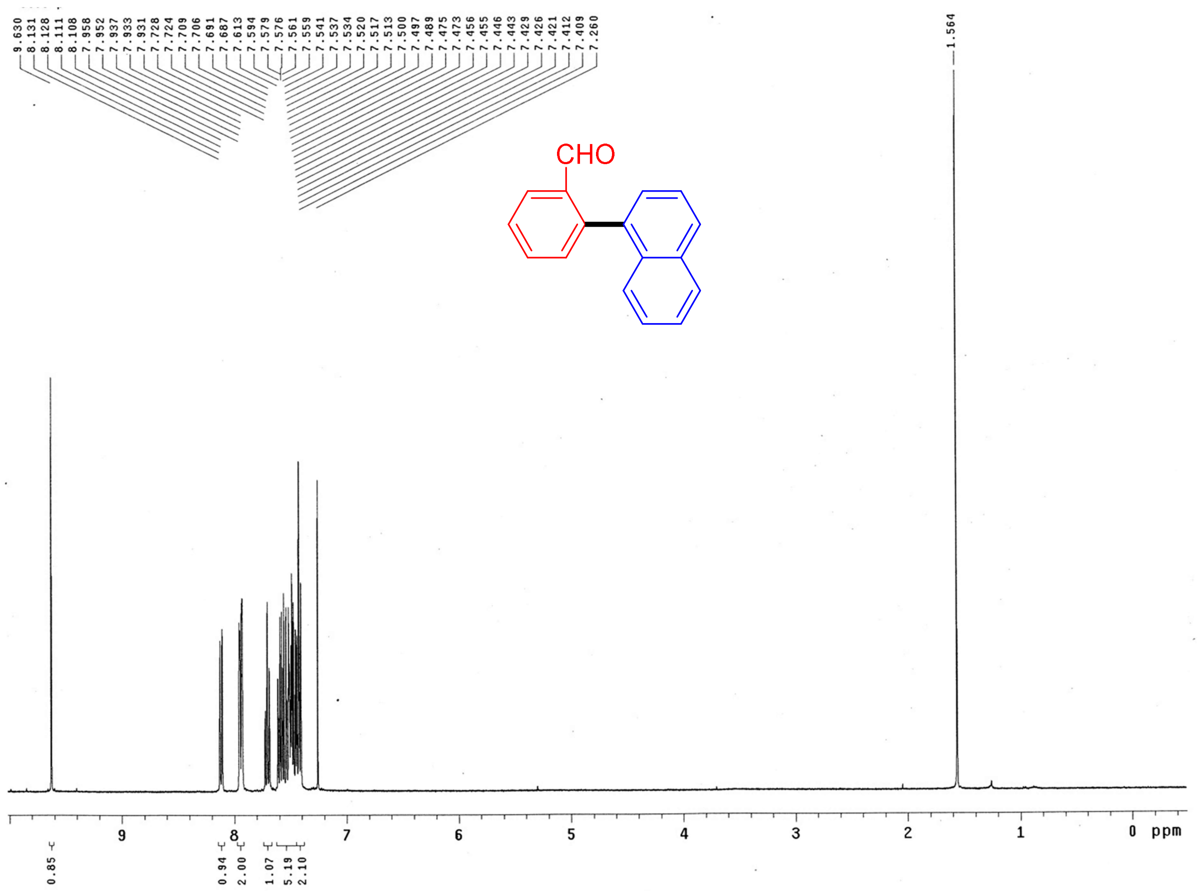

^{13}C NMR ($CDCl_3$, 100 MHz) spectrum of compound **5pb**

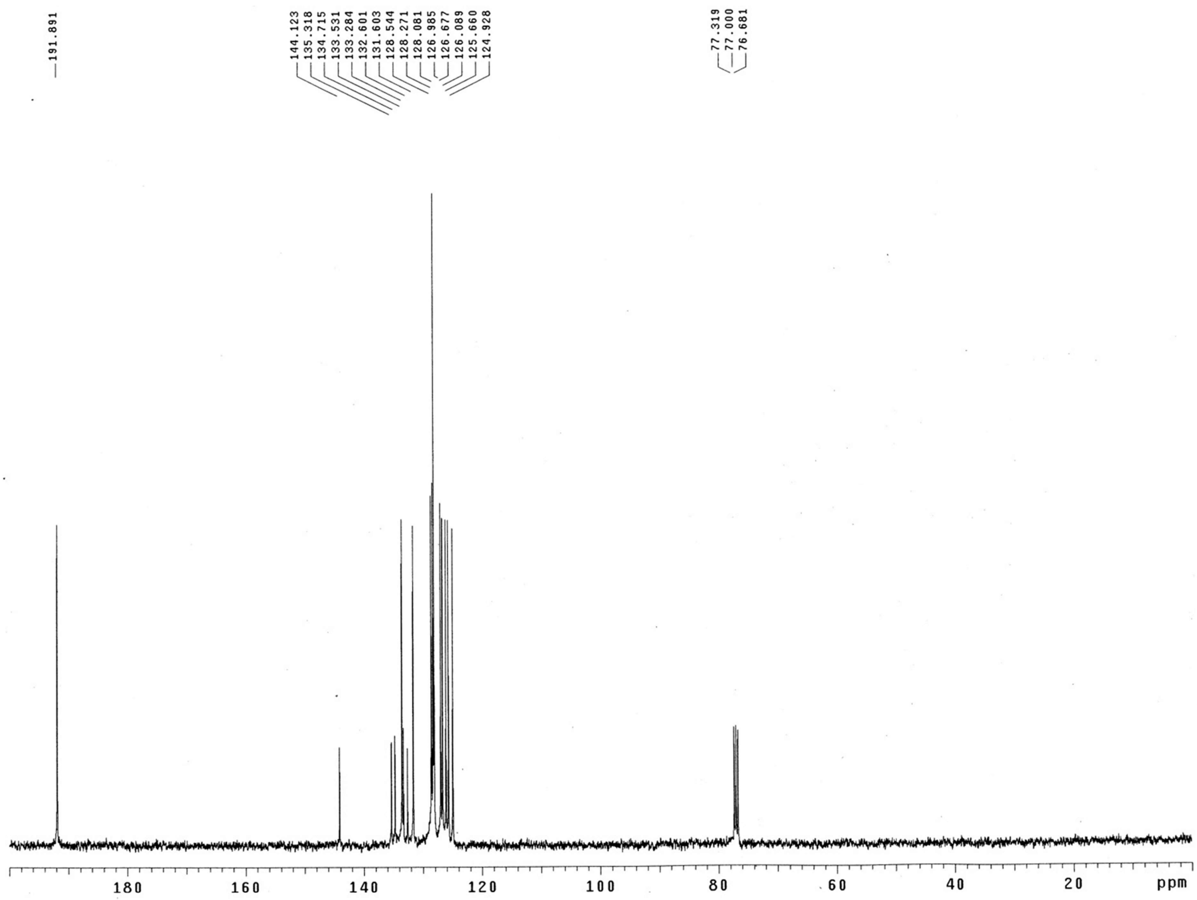

^{1}H NMR ($CDCl_3$, 400 MHz) spectrum of compound **5kb** (table 3)

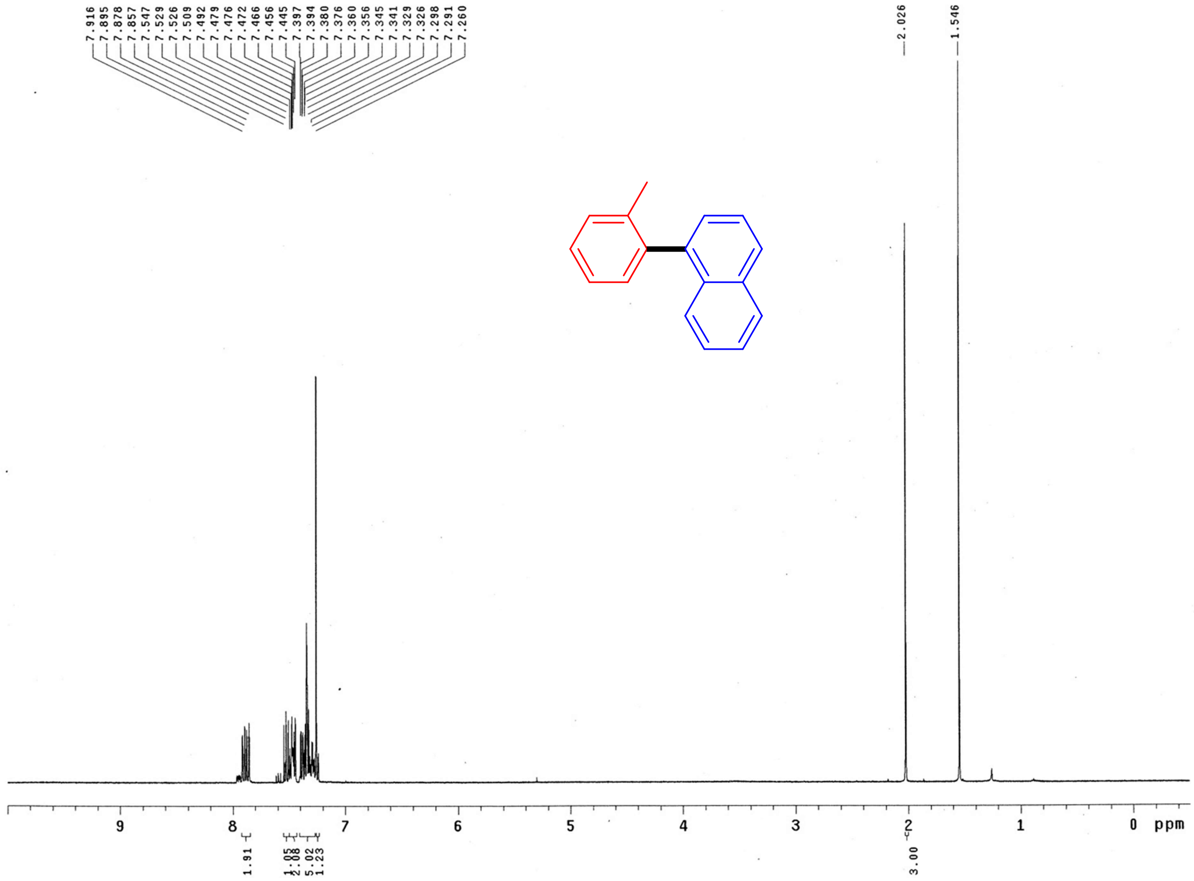

^{13}C NMR ($CDCl_3$, 100 MHz) spectrum of compound **5kb**

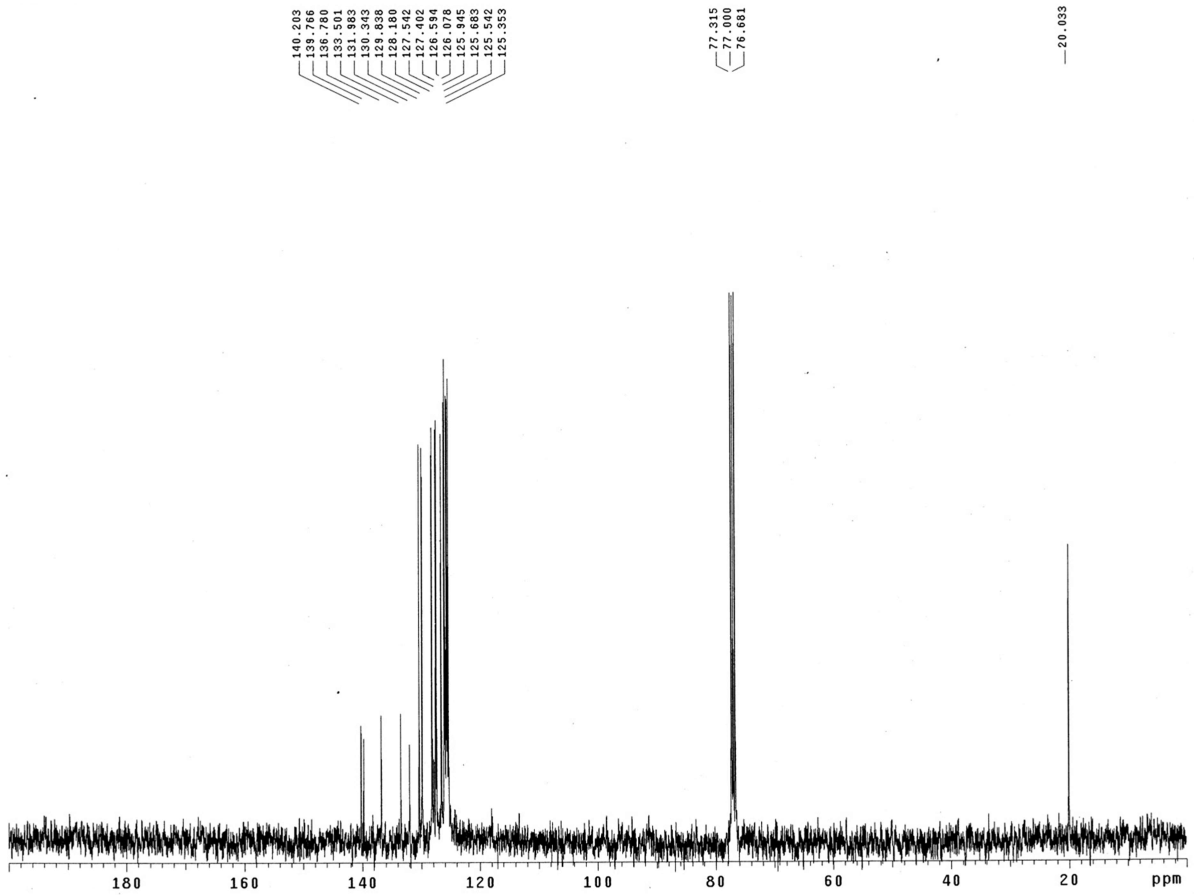

^{1}H NMR ($CDCl_3$, 400 MHz) spectrum of compound **5ob** (table 3)

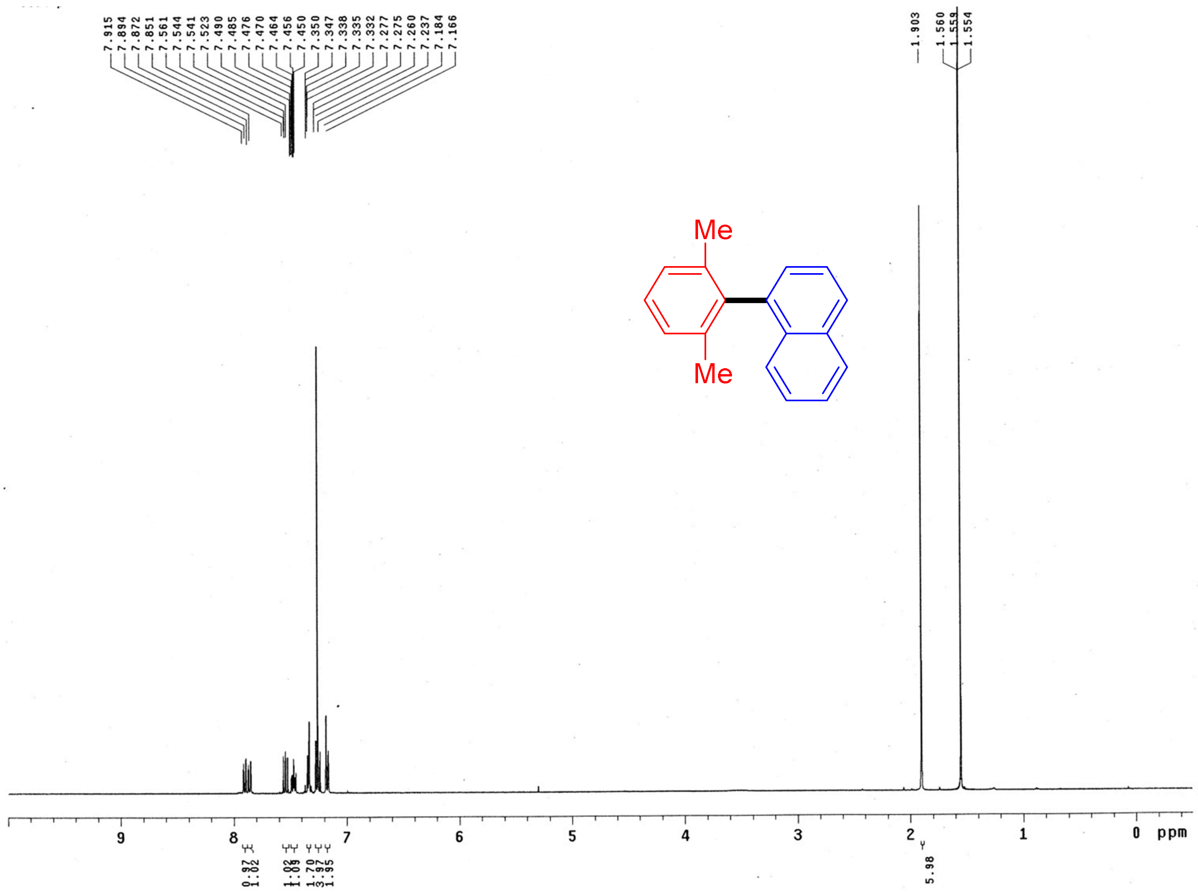

^{13}C NMR ($CDCl_3$, 100 MHz) spectrum of compound **5ob**

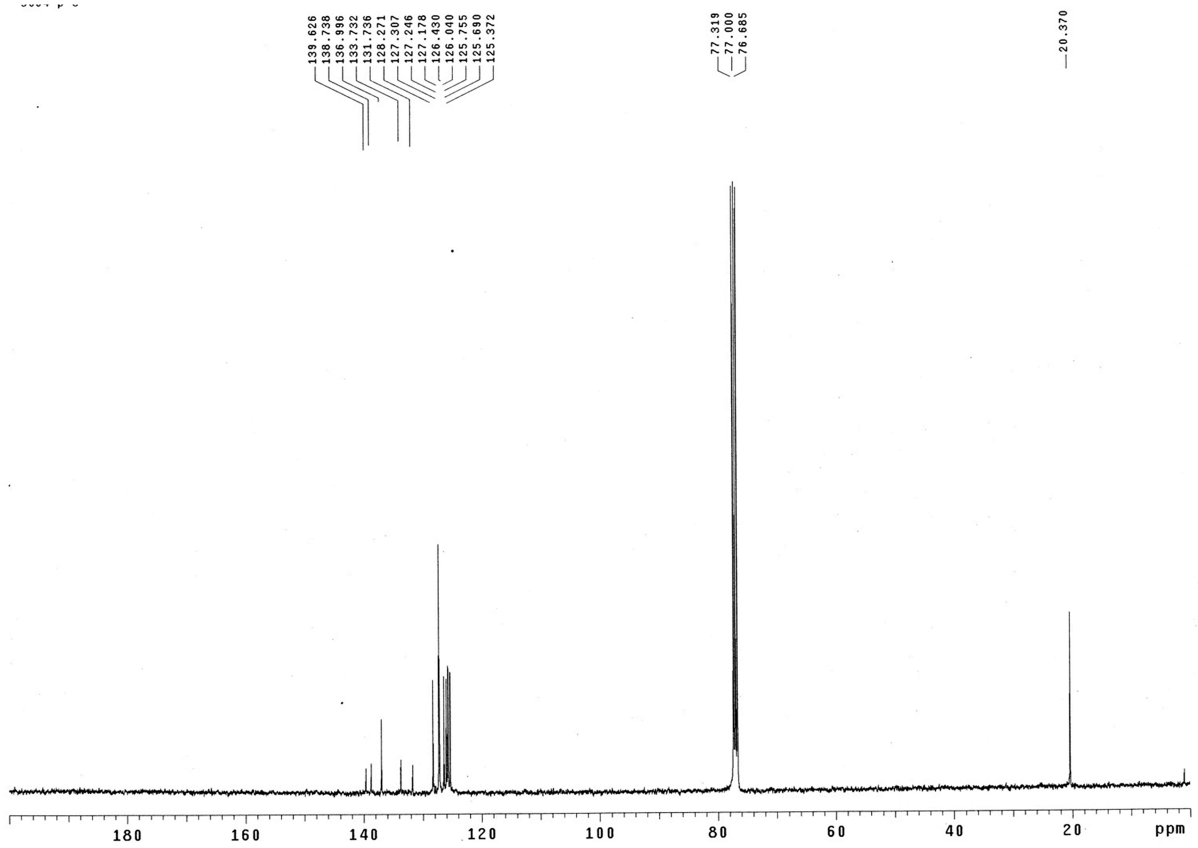

^{1}H NMR ($CDCl_3$, 400 MHz) spectrum of compound **5od (=5kc)** (table 3)

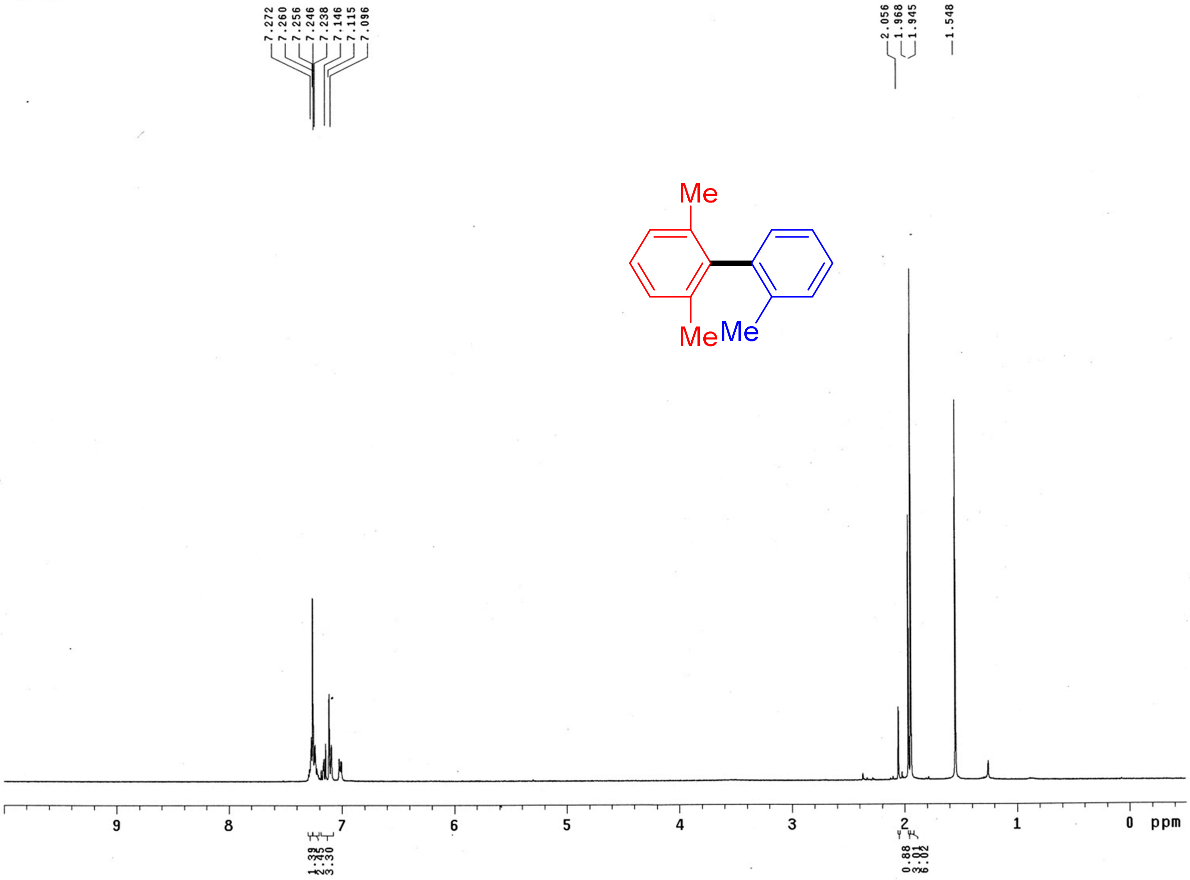

^{13}C NMR ($CDCl_3$, 100 MHz) spectrum of compound **5od (=5kc)**

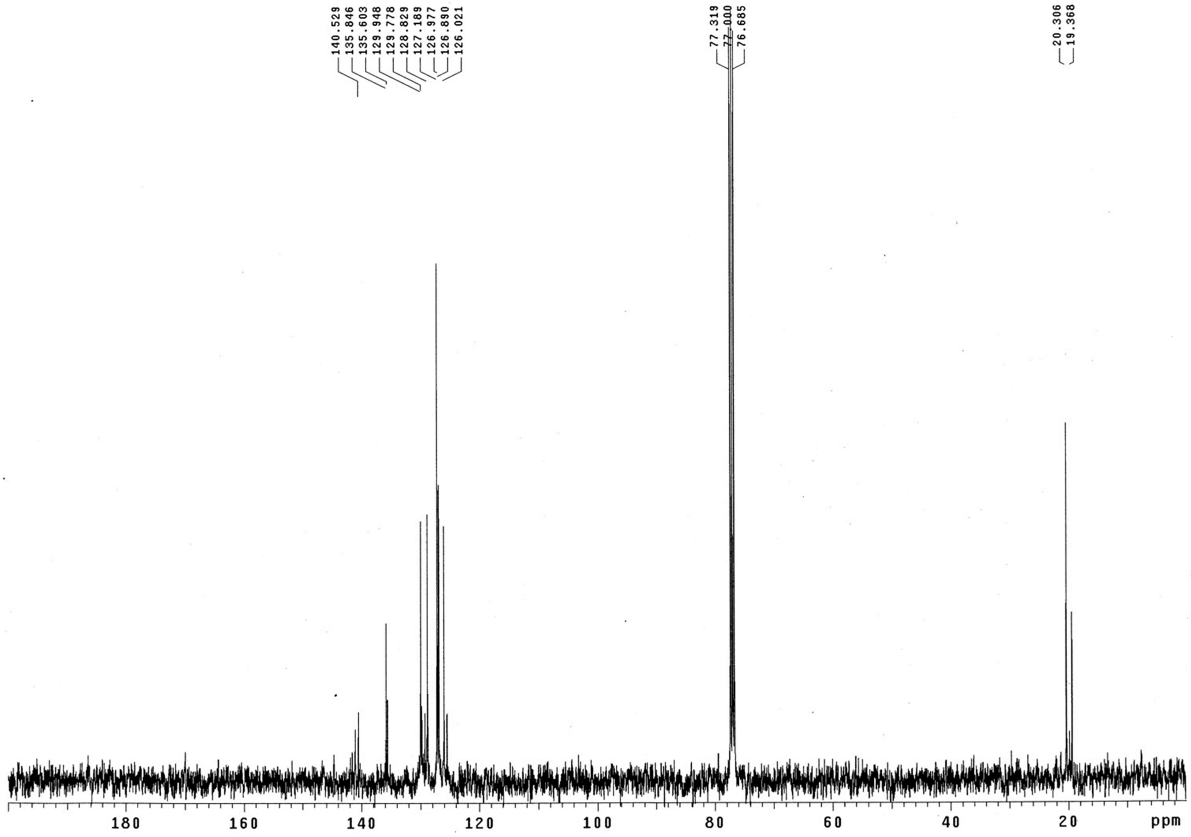

^{1}H NMR ($CDCl_3$, 400 MHz) spectrum of compound **5nb** (table 3)

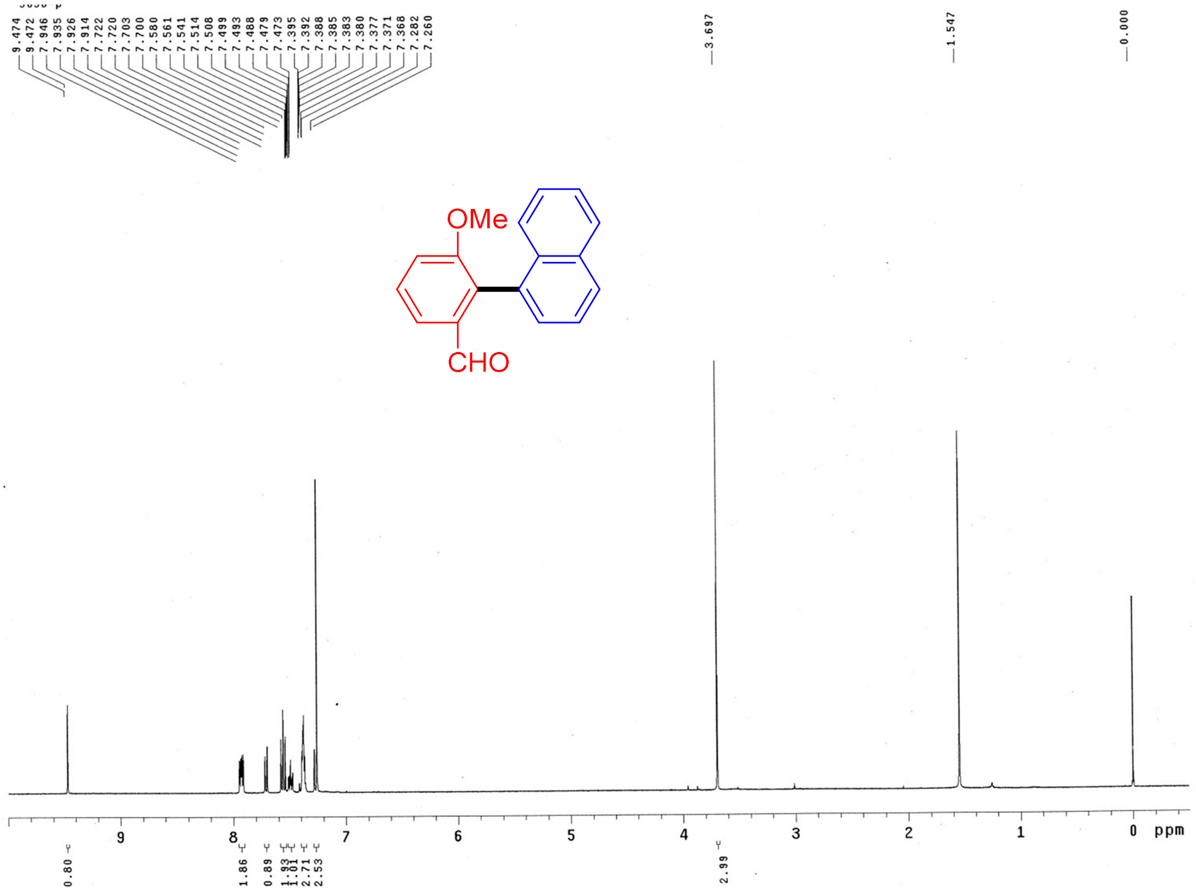

^{13}C NMR ($CDCl_3$, 100 MHz) spectrum of compound **5nb**

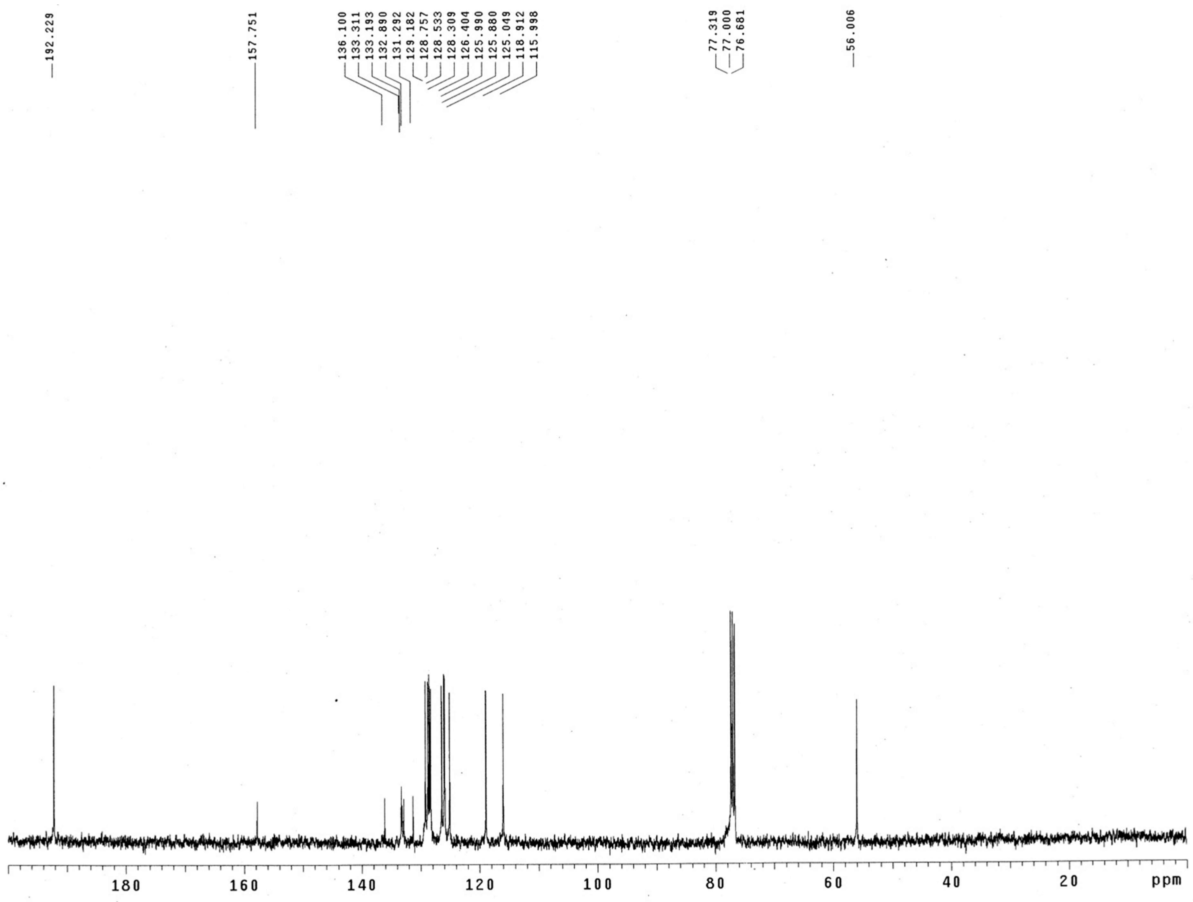

^{1}H NMR ($CDCl_3$, 400 MHz) spectrum of compound **5ne** (table 3)

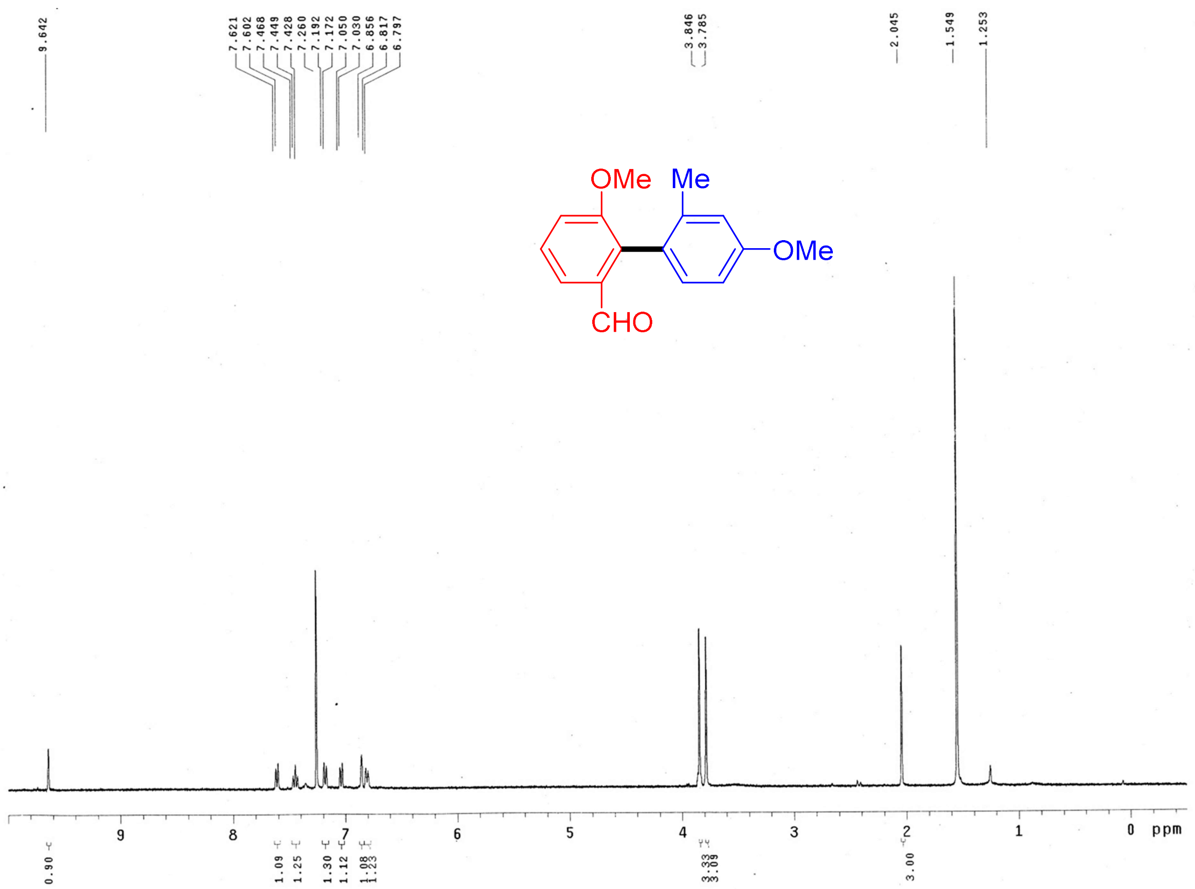

^{13}C NMR ($CDCl_3$, 100 MHz) spectrum of compound **5ne**

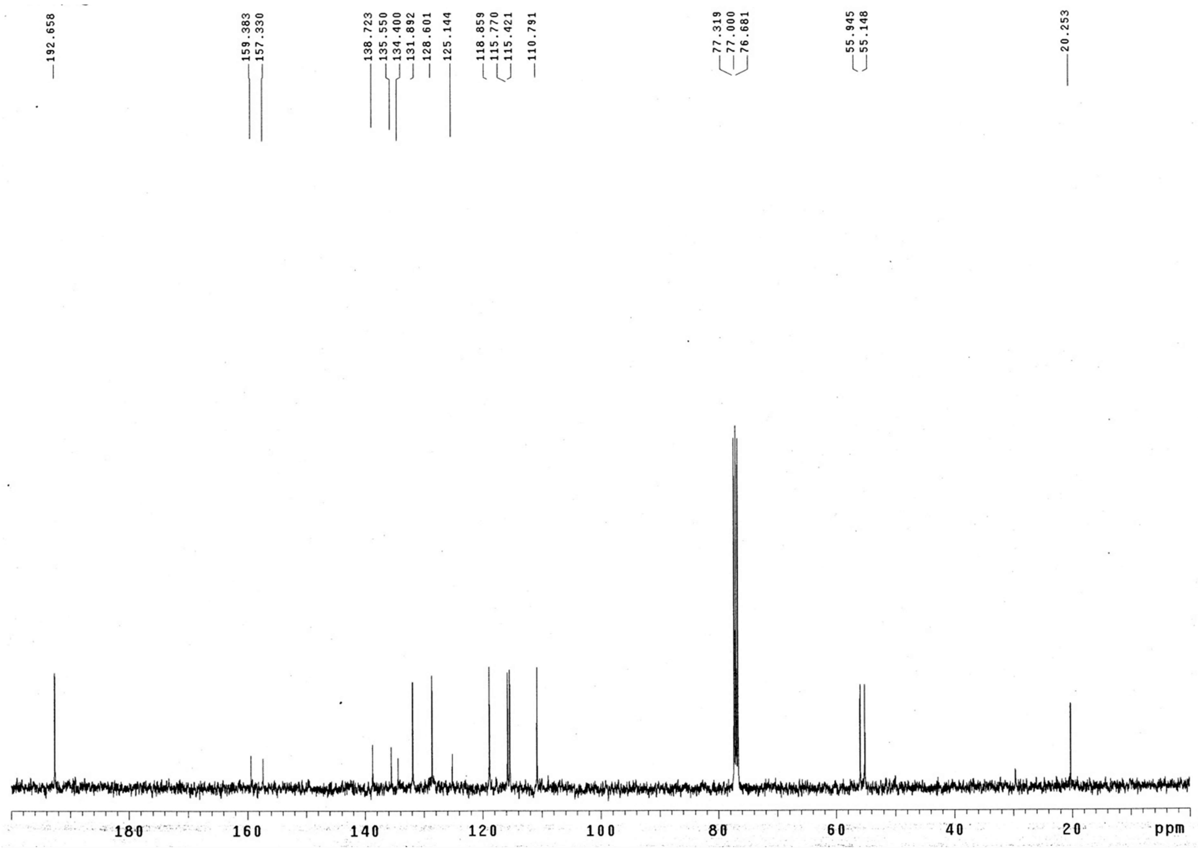

^{1}H NMR ($CDCl_3$, 400 MHz) spectrum of compound **5nf** (table 3)

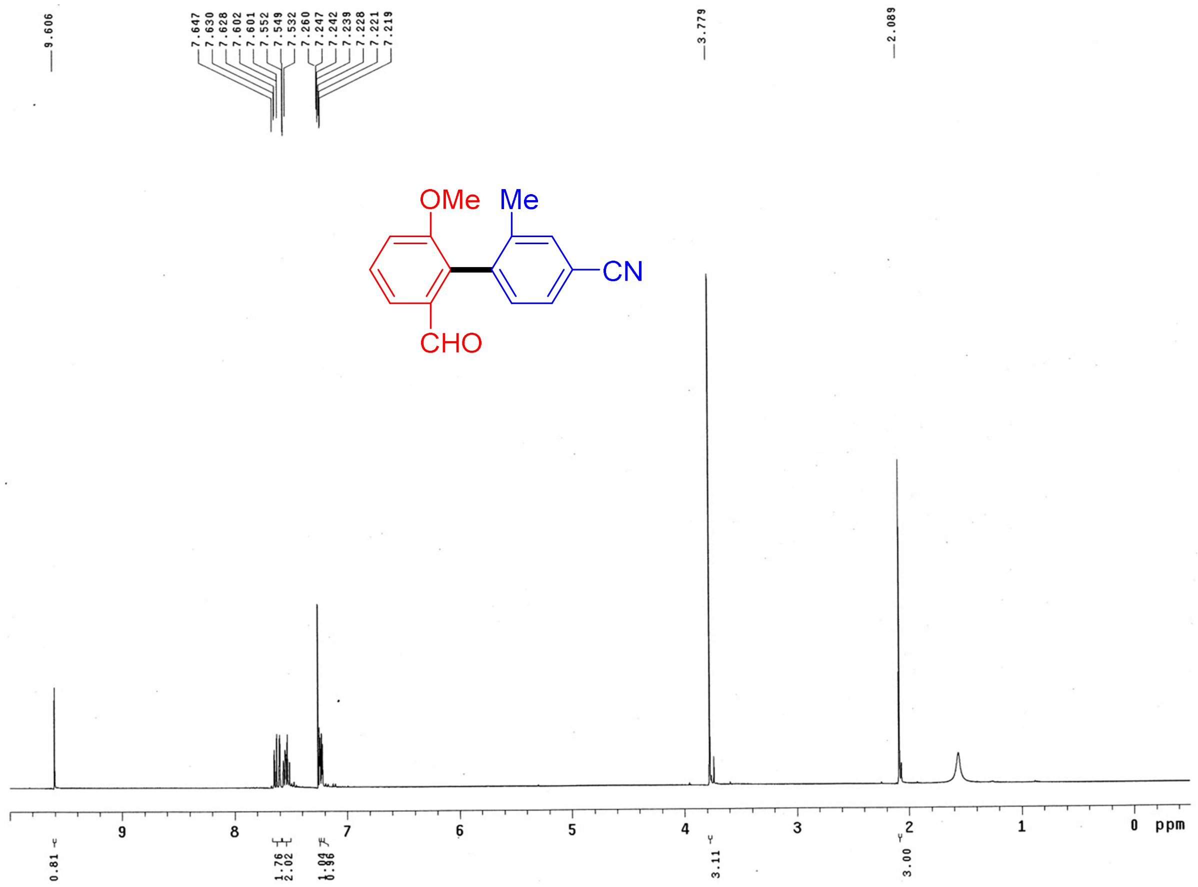

^{13}C NMR ($CDCl_3$, 100 MHz) spectrum of compound **5nf**

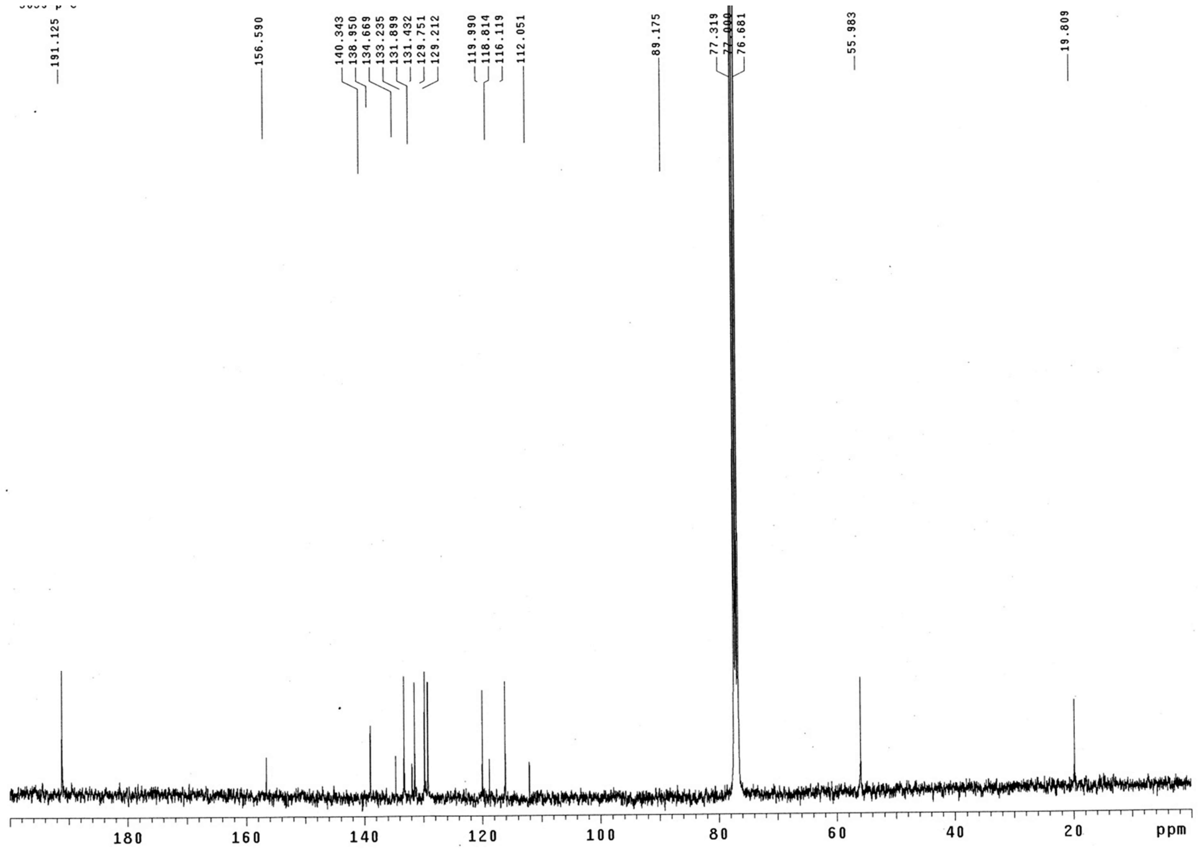

Supplement: Supplementary file 1 [file molecules-26-06703-s001.zip › molecules-1438411-supplementary.pdf]
